# Modified Lipid Extraction Methods for Deep Subsurface Shale

**DOI:** 10.3389/fmicb.2017.01408

**Published:** 2017-07-25

**Authors:** Rawlings N. Akondi, Ryan V. Trexler, Susan M. Pfiffner, Paula J. Mouser, Shikha Sharma

**Affiliations:** ^1^Department of Geology and Geography, West Virginia University Morgantown, WV, United States; ^2^Civil, Environmental and Geodetic Engineering, The Ohio State University Columbus, OH, United States; ^3^Center for Environmental Biotechnology, University of Tennessee Knoxville, TN, United States

**Keywords:** PLFA, DGFA, microbial biomass, deep subsurface, shale ecosystem

## Abstract

Growing interest in the utilization of black shales for hydrocarbon development and environmental applications has spurred investigations of microbial functional diversity in the deep subsurface shale ecosystem. Lipid biomarker analyses including phospholipid fatty acids (PLFAs) and diglyceride fatty acids (DGFAs) represent sensitive tools for estimating biomass and characterizing the diversity of microbial communities. However, complex shale matrix properties create immense challenges for microbial lipid extraction procedures. Here, we test three different lipid extraction methods: modified Bligh and Dyer (mBD), Folch (FOL), and microwave assisted extraction (MAE), to examine their ability in the recovery and reproducibility of lipid biomarkers in deeply buried shales. The lipid biomarkers were analyzed as fatty acid methyl esters (FAMEs) with the GC-MS, and the average PL-FAME yield ranged from 67 to 400 pmol/g, while the average DG-FAME yield ranged from 600 to 3,000 pmol/g. The biomarker yields in the intact phospholipid Bligh and Dyer treatment (mBD + Phos + POPC), the Folch, the Bligh and Dyer citrate buffer (mBD-Cit), and the MAE treatments were all relatively higher and statistically similar compared to the other extraction treatments for both PLFAs and DGFAs. The biomarker yields were however highly variable within replicates for most extraction treatments, although the mBD + Phos + POPC treatment had relatively better reproducibility in the consistent fatty acid profiles. This variability across treatments which is associated with the highly complex nature of deeply buried shale matrix, further necessitates customized methodological developments for the improvement of lipid biomarker recovery.

## Introduction

The microbial ecology of the deep subsurface ecosystem has received increased research attention over the last two decades (e.g., Fredrickson et al., 1997; Krumholz et al., 1997; Onstott et al., 1998; Whitman et al., 1998; D'hondt et al., 2004; Biddle et al., 2006; Fredricks and Hinrichs, 2007; Pfiffner et al., 2006; Schippers and Neretin, 2006; McMahon and Parnell, 2014; Inagaki et al., 2016), with some studies suggesting that the deep subsurface biosphere contributes as much as 50% of the Earth's biomass (Whitman et al., 1998; McMahon and Parnell, 2014). Consequently, the role of deep subsurface microbial communities has become increasingly important. Energy and environmental applications of black shales have also induced research interests on the microbial functional diversity in the deep subsurface shale ecosystem. Unconventional hydrocarbon production in black shales through hydraulic fracturing (Rogner, 1997; Curtis, 2002; Passey et al., 2010; Chengzao et al., 2012), has bolstered the possibility of introducing exogenous microbes which could alter the microbial community structure of the deep subsurface shale ecosystem. Accordingly, isotopic evidence of potential biogenic gas production in the Marcellus Shale (Sharma et al., 2014) and the presence of microbial signatures in produced fluids from hydraulically fractured wells (Mohan et al., 2013; Cluff et al., 2014; Gaspar et al., 2014) has further intensified the significance of microbial activities in relation to the shale ecosystem and energy applications. While unconventional hydrocarbon production has the potential of altering the deep subsurface shale ecosystem, deep subsurface microbial activity can also influence the hydrocarbon production potential and efficiency. For example, microbial metabolites can interfere negatively with hydrocarbon production by clogging hydraulically fractured formations, corroding wells, and increasing H_2_S content (gas souring, Gaspar et al., 2014) while also improving shale gas production potential through microbial enhanced oil recovery (Lazar et al., 2007). Thus, the study of microbial community dynamics of deeply buried subsurface shale ecosystem becomes very essential.

Despite evidence of endogenous microbial life in the deep subsurface, the numerous challenges involved in isolating and culturing deep subsurface microbes makes it difficult to actually characterize *in situ* subsurface microbial communities. One molecular tool that provides a sensitive measure of *in situ* biomass density is the microbial lipid analysis. The ester-linked phospholipid fatty acid (PLFA) is commonly used in measuring viable biomass and characterizing microbial cumminuty structure (White et al., 1979). Upon microbial cell death, the membrane phospholipid in the PLFA breaks down leading to the formation of diglyceride fatty acid (DGFA; Kieft et al., 1994; White and Ringelberg, 1998). Thus, the phospholipid fatty acids (PLFAs) provide a sensitive molecular-based estimation of the contemporary viable microbial community and the diglyceride fatty acids (DGFAs) provide an estimate of the non-viable microbial community (Kieft et al., 1994; Haldeman et al., 1993; White and Ringelberg, 1998; Fredrickson et al., 1997; Ringelberg et al., 1997). Combined, these measurements convey information on the relationship between the viable and non-viable biomass as well as shed some insight into community composition, nutritional status, and other environmental stressors.

Even though lipid analysis is a very sensitive method, the informative quality of the technique can be reduced by low lipid concentrations and variations in matrix property (Gómez-Brandón et al., 2008). Therefore, low microbial biomass and ineffective extractions will generate unreliable results. Many procedures have been developed and modified to improve the extraction of the microbial lipids from various matrices (Bligh and Dyer, 1959; Christie, 1993; Brinch-Iversen and King, 1990; Nielsen and Petersen, 2000; Cequier-Sánchez et al., 2008). One of the most used lipid extraction methods, especially for extraction from environmental samples, is the Bligh and Dyer single-phase extraction method (e.g., Bligh and Dyer, 1959; White et al., 1979; Guckert et al., 1985; Frostegard et al., 1991; Kieft et al., 1994; Fredrickson et al., 1997; Ringelberg et al., 1997; White and Ringelberg, 1998; Pfiffner et al., 2006). Contemporary instrumental methods have also brought about modifications to lipid extractions which have gone a long way to improving yields. Some of these methods include the use of pressurized or accelerated solvent extraction and microwave irradiation or ultrasonication (Vetter et al., 1998; Batista et al., 2001; Young, 1995; Lores et al., 2006; Gómez-Brandón et al., 2008). Furthermore, other lipid improvement methods have been developed to optimize the recovery of ether-linked microbial lipid biomarkers (Zhang et al., 2003; Lengger et al., 2012).

While many modifications have been made on lipid analysis for samples of various matrices, the unique properties of deeply buried shale necesitates that current extraction procedures should also be optimized in an effort to generate high quality results. Shales are characterized by complex organic matter matrix, mineralogy, and chemistry which can impede efficient lipid extraction (Shaw and Weaver, 1965; Boles and Franks, 1979; Aplin and Macquaker, 2011; Chermak and Schreiber, 2014). Clay colloids in the shale sediments also bind to the lipids, interfering with adequate lipid recovery. Additionally, the small pore sizes, low permeability (Colwell et al., 1997; Fredrickson et al., 1997; Onstott et al., 1998; Javadpour, 2009; Sondergeld et al., 2010), and overall low biomass density (Fredrickson et al., 1997) associated with the deep subsurface shale environment may also hinder lipid extraction. More so, the extreme difficulty associated with preventing potential exogenous microbial contamination during drilling and processing of subsurface cores (Wilkins et al., 2014) also hampers molecular analysis of deep subsurface microbial communities. Given the inaccessibility of the deep surface environment and the high economic cost associated with well drilling, maximizing microbial scientific output from the already rare and precious samples becomes critical.

In this study, we seek to improve the recovery of microbial biomass and diversity for deep subsurface shale matrices with low biomass densities through the analysis of ester-linked microbial lipid biomarkers. Lipid extraction experiments based on modifications of previous extraction procedures were carried out to examine the combination of different extraction solvents, buffers, and biochemical amendments for both PLFAs and DGFAs. Three extraction methods were tested: (i) modified Bligh and Dyer (mBD), (ii) Folch (FOL), and (iii) microwave-assisted extraction (MAE) treatments. Within the mBD method, modifications based on phosphate (White et al., 1979) or citrate buffer (Frostegard et al., 1991) were utilized. The effectiveness of three different biochemical amendments; (i) magnesium (Mg^2+^), (ii) *Escherichia coli* biomass (*E. coli*), and (iii) 1-palmitoyl-2-oleoyl-sn-glycero-3-phosphocholine (POPC) were evaluated on their ability to enhance the yield and profile quality of the standard modified Bligh and Dyer phosphate (mBD + Phos) treatments. All samples used are deep subsurface shale samples cored from ~7,000 ft. and the resultant lipids from all extraction treatments were transesterified into fatty acid methyl esters (FAMEs) and analyzed by gas chromatography-mass spectrometry (GC-MS).

## Methods

**Figure 1:**
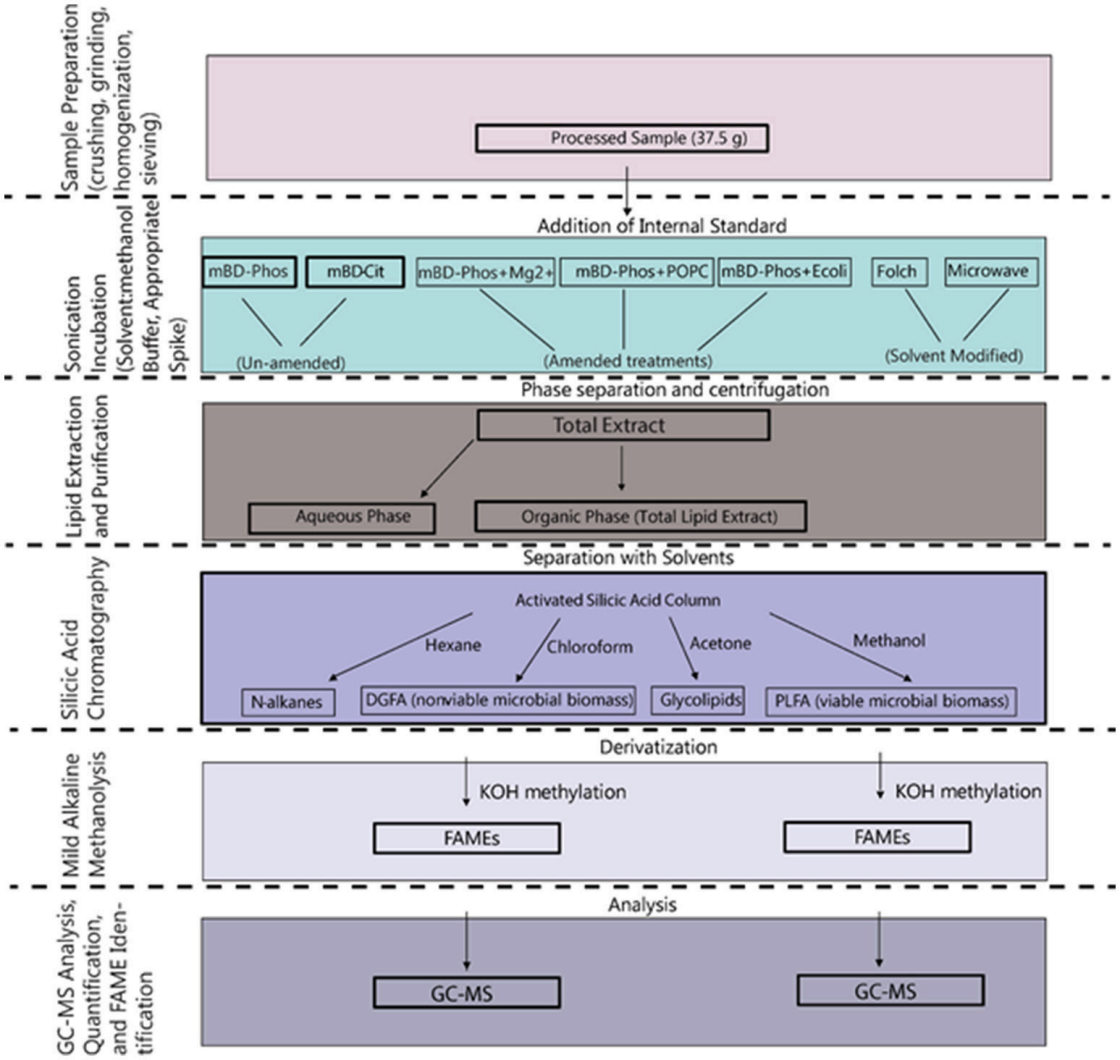
Schematic overview of the procedures involved in the extraction and methylation of the lipid fatty acids.

A summarized scheme of the methodology for the lipid extraction including experimental treatments is shown in Figure 1. All extraction treatments and their reagents are also listed in Table 1.

**Table 1:** Representation of reagents and materials used in the various extraction treatments.

**Extraction type**	**Extraction conditions**	**Treatment name**
Modified Bligh and Dyer	Phosphate Buffer	mBD-Phos
	Citrate Buffer	mBD-Cit
	Phosphate Buffer + Mg^2+^	mBD-Phos + Mg^2+^
	Phosphate Buffer + POPC	mBD-Phos + POPC
	Phosphate Buffer + *E. coli*	mBD-Phos + *E. coli*
Modified Folch Extraction	Chloroform: Methanol	Folch
Microwave Assisted Extraction	Chloroform: Water	Microwave

*Seven treatment conditions were each tested in triplicates (n = 20) except the mBD + Phos treatment which was done in duplicate. Blanks were analyzed for each extraction treatment*.

### Reagents and materials

Critical analytical precautions were taken to ensure that materials and reagents were free of organic contaminants. All reagents and solvents used during the extraction and analytic experimental process were of purest grade (HPLC, Fisher Optima). Glassware were cleaned in a 10% (v/v) micro alkaline cleaning solution (International Products Corporation, Burlington, NJ) and rinsed with 70% methanol, 5 times with distilled water, and 5 times with Millipore water. All glassware and tools were autoclaved at 550°C. Metal lab wares (forceps, mortar, pestle, and spatulas) were cleaned with tap water, distilled water, and finally with a solution of 1:1 chloroform:methanol. Teflon-lined caps were cleaned in the same manner as the glassware and then solvent rinsed with acetone. Procedural blanks were also included in each extraction treatment to monitor laboratory contamination. With the exception of standard peaks, blanks did not have any FAME peaks. Internal standards of different concentrations (1, 5, 10, 20, 30, 40, and 50 pmol/μL) were prepared and analyzed on the GC-MS to determine the detection limit and to also establish the best sample dilution range. The standard curve and the regression analysis had a linear relationship (0.99). Based on the lowest dilution concentration, the detection limit for the GC-MS was 1 pmol/uL.

#### Sample preparation and extraction procedures

Non-pristine core samples taken from commercial production wells at ~7,000 ft in the Marcellus Shale in Pennsylvania and West Virginia were crushed using a sterile mortar and pestle and homogenized by stirring thoroughly (Thomas Scientific, Swedesboro, NJ). We took great care to ensure sample homogeneity by: (1) paring the outer portion of rock, ensuring any handling/storage effects were minimized; (2) crushing cores using a sterile mortar and pestle; and (3) homogenizing the samples by first stirring thoroughly then transferring the crushed samples to muffled aluminum foils and continuing to homogenize by folding, dividing, and mixing different corners of the sheet. The homogenized crushed samples were passed through a sterile brass sieve series (Dual Manufacturing Co., Franklin Park, IL), where we retained only the crushed core that passed a 500 μm screen. After homogenization, the samples were then partitioned for subsequent extractions. Lipid extractions and analyses were performed at the Center for Environmental Biotechnology at the University of Tennessee (Knoxville, TN, USA).

### Extraction

#### Modified Bligh and Dyer (mBD) method

Samples for the mBD treatments (*n* = 14) were extracted by the Bligh and Dyer procedure (Bligh and Dyer, 1959), with modifications using phosphate buffer (mBD + Phos; *n* = 11) as described in White et al. (1979) and citrate buffer (mBD + Cit; *n* = 3) as described in Frostegard et al. (1991). The following materials were used as amendments in the phosphate buffered treatments (mBD + Phos; *n* = 9) to test their suitability for optimizing recovery:

##### Escherichia coli (n = 3)

Stock solutions for the *E. coli* amendment were prepared by streaking Luria Broth agar plates with an *E. coli* aliquot and grown overnight at 37°C to isolate colonies. A colony was then picked and cultured in Luria Broth liquid for 16 h at 37°C. Cells were counted by hemocytometers under light microscopy and via the EMD Millipore Guava Flow Cytometer (Billerica, MA). Cells were diluted to 1 × 10^5^ cells/mL using 1X phosphate buffer saline solution. 1 mL of the resulting stock solution was then added to each of the extraction mixtures for the phosphate-buffered *E. coli* treatments (mBD + Phos + *E. coli*).

##### Intact phospholipid (n = 3)

Stock solutions of 3.3 × 10^−5^ mol/mL of 1-palmitoyl-2-oleoyl-sn-glycero-3-phosphocholine (POPC) were diluted with chloroform to a working solution concentration of 3.3 × 10^−12^ mol/mL. 1 mL of the solution was added to each of the extraction mixtures for the phosphate-buffered POPC treatments (mBD + Phos + POPC). The concentrations of the *E. coli* and POPC amendments were determined based on the PLFA-to-biomass conversion factor of 1.4 × 10^−17^ mol PLFA/cell (Frostegard and Baath, 1996).

##### Magnesium chloride (n = 3)

The magnesium (Mg^2+^) amendment was prepared by adding 4.767 g of magnesium chloride hexahydrate (MgCl_2_ 6H_2_O) to a 100 mL aliquot of prepared phosphate buffer. 30 mL of the phosphate buffer-magnesium solution was then added to the extraction mixture to yield a final concentration of 1,200 ppm Mg^2+^ in each of the extraction mixtures for phosphate-buffered Mg^2+^ treatments (mBD + Phos + Mg^2+^).

Lipid extractions were carried out from 37.5 g of the crushed rock. The crushed rock samples were transferred to a 250 mL glass centrifuge bottle and then suspended in solvent extraction mixtures of chloroform-methanol-appropriate buffer, 1:2:0.8 (v/v/v, Chloroform: MeOH: Buffer). The concentration and pH of the buffers were as follows; citrate (0.15 m, pH 4.0) and phosphate (0.05, pH 7.4). The appropriate amendments were then added to the phosphate buffered treatments (*n* = 9, mBD-Phos + *E. coli*, mBD-Phos + Mg^2+^, and mBD-Phos + POPC). The remaining phosphate buffered (*n* = 2, mBD-Phos) and citrate buffered (*n* = 3, mBD-Cit) samples were extracted without an exogenous amendment to further compare the performance of amended samples to the un-amended extracted samples. Due to the implication of reproducibility to the extraction of microbial biomass, each extraction treatment was done in triplicates. 50 uL of 50 pmol/ μL of internal standard (1, 2-dinonadecanoyl-sn-glycero-3-phosphocholine, Avanti Polar Lipids) was added to each treatment. The suspension was shaken and sonicated two times in an ultrasonicator for 30 to 45 s with a 30 s interval between sonication cycles. The internal standard was used to measure extraction efficiency of the lipids. Bottles were shaken for 15 s and vented before incubation overnight in the dark at room temperature. After incubation, samples were held at 4°C and centrifuged for 30 min at 2,000 rpm. The resulting supernatant was transferred to a 250 mL separatory glass funnel. Chloroform and water were added to the suspension (1:1:0.9, chloroform: methanol: buffer v/v/v) and the separatory funnels were shaken for 15 s and left to rest overnight to split phase (upper: aqueous and lower: organic containing the lipids). While the lipids were kept in the separatory funnel to separate phase, the already extracted shale samples were re-extracted with same solvents and reagents. Re-extraction allowed fresh solvent to contact and penetrate new surface area in the shale. After separation, the organic phase was collected into a 250 mL round bottom flask and evaporated to near dryness using a rotavap system (Buchi Corporation, New Castle, DE). The total lipid extract (TLE) was then quantitatively transferred into test tubes using three washes of 2 mL of chloroform, after which the solvent was evaporated with an N_2_ blowdown evaporator at 37°C. The dried total lipid extracts (TLEs) were resuspended in 2 mL of chloroform and stored for silicic acid chromatography.

#### Modified Folch method

Apart from the modifications below, the Folch samples (*n* = 3) were extracted with same extraction mixtures and procedure as described by Folch et al. (1957). Due to volume constraints in maintaining a 20:1 ratio of solvent:sample with 37.5 g of homogenized shale, each sample was divided into 4 round bottom flasks during solvent incubation. For each sample subset, 125 mL of chloroform, 62.5 mL of methanol, and 9.375 g of homogenized shale were added to provide a ratio of 2:1 chloroform:methanol (v/v). The organic fraction from each subset was fractionated and stored for silicic acid chromatography and subsequent trans-methylation.

#### Microwave assisted extraction (MAE) method

The MAE samples (*n* = 3) also had volume constraints, and as such, each sample was divided initially into 7 subsamples. The solvent for the MAE was chloroform:methanol rather than hexane:acetone which are the most frequently used solvents in MAE (Lopez-Avila et al., 1994; Lopez-Avila, 1999; Gómez-Brandón et al., 2008, 2010). Our modification was based on the effectiveness of chloroform:methanol as reagent solvent mixtures for lipids from environmental samples (Ewald et al., 1998; Renaud et al., 1999). To create a 9:1 (v/v) ratio of chloroform:methanol, 48.2 mL of chloroform, 5.35 mL of methanol, and 5.35 g of homogenized shale were added to each Teflon reaction vessel. The vessels were irradiated in a Milestone Ethos EX Microwave Extractor System (Milestone Inc., Shelton, CT) with a temperature ramp of 2.5 min (2,450 mHz, 630 W, 100°C max temperature) and held for an additional 2.5 min (2,450 mHz, 630 W, 100°C max temperature). Stir-bars were engaged during the irradiation period and vessels were allowed to cool down for 15 min before pouring the contents into 250 mL round bottom centrifuge bottles. Samples were centrifuged and transferred to separatory funnels as described for mBD samples. Once in separatory funnels, 150 mL of water was added to break phase. The samples were shaken for 15 s and allowed to rest overnight to separate phases entirely. The resulting organic fraction was fractionated and stored for silicic acid chromatography and subsequent trans-methylation.

### Separation

#### Silicic acid chromatography (SAC)

The extracted lipids were fractionated on an activated silicic acid column, 100–200 mesh powder (dried at 110°C for 1 h; Clarkson Chromatography Products, Inc), into fractions of different polarities using hexane, chloroform, acetone, and methanol. The silicic acid columns were constructed by loading a suspension of 0.5 g of silicic acid in 5 mL of hexane on to glass pipettes. Prior to loading the silicic acid column, glass wool was placed at the bottom of the pipettes and rinsed with 2 mL of hexane. After loading the column with silicic acid slurry, sodium sulfate (Na_2_SO_4_) was added to the top of the column to exclude the possibility of the presence of oxygen. The TLE was then suspended in 200 μL of hexane and loaded onto the top of the silicic acid column. We repeated this quantitative transfer three times and care was taken to not disturb the surface of the column once the sample was loaded. A series of four solvents of increasing polarity were then used to separate the lipid classes: hydrocarbons = 5 mL of hexane, neutral lipids = 5 mL of chloroform, glycolipids = 5 mL of acetone, polar lipids = 10 mL of methanol into test tubes. We maintained silicic acid and solvent ratio of 1:10 (g silicic acid: mL eluting solvent), except for methanol. The resulting chloroform fraction was methylated into fatty acid methyl esters (FAMEs) by mild alkaline methanolysis and analyzed for DGFA while the methanol fraction was methylated and analyzed for PLFA (White et al., 1979; Guckert et al., 1985; Kieft et al., 1994; Ringelberg et al., 1997; White and Ringelberg, 1998).

### Analysis

#### GC-MS analysis, quantification, and FAME identification

Lipid samples were then dissolved in 200 μL of hexane containing 50 pmol/uL of external injection standard (docosanoic acid methyl ester; Matreya, Inc) and transferred into GC-MS vials containing 500 μL glass inserts. The external standard was used to calculate the peak area of the FAME profiles. Aliquots of samples were then injected into an Agilent 6,890 series gas chromatograph interfaced to an Agilent 5,973 mass selective detector equipped with a non-polar cross-linked methyl silicone column (Restek RTX-1 column 60 m, 0.25 mm I.D. × 0.25 μm film thickness) to be further separated, identified, and quantified. The gas chromatography operating conditions were as follows: 60°C for 2 min then ramped at a rate of 10°C/min to 150°C and followed by a second ramp at 3°C/min to 312°C for a total run time of 65 min (White and Ringelberg, 1998). The injector temperature was 230°C; the detector temperature was 300°C; and Helium was the carrier gas. The PLFA standards methyl ester mixtures, Bacterial Acid Methyl Esters CP Mixture, BacFAME (1114); and Polyunsaturated FAME Mixtures, PUFA-2(1081); and PUFA-3 (1177) (Matreya LLC, State College, Pennsylvania, USA) were included in each sample run to calibrate retention times and assist with peak identification. All identified peaks were confirmed across all samples and validated independently via GC-MS spectra using the Agilent MSD ChemStation Data Analysis Software F.01.00 along with the NIST11 compound library. All identified peaks were confirmed across all samples and validated independently via GC-MS spectra confirmation. FAME identities were as described in Ringelberg et al. (1989).

To validate the proportional relationship, a regression analysis of external standard concentrations and peak areas from the standard curve samples demonstrated a linear relationship (*R*^2^ > 0.99) in the scope of 1–50 pmol/uL. However, in some PLFA samples, the external standard peak co-eluted with a minor abundance of phthalate isomers and was corrected before the FAME concentration was calculated. We used 24 samples of unadulterated external standard peaks (50 pmol/uL) to adjust the total ion concentration (TIC) of the PLFA samples that contained the phthalate isomers. The area of the 10 largest base peaks (74, 75, 87, 111, 129, 143, 199, 255, 311, and 354 m/z) of docosanoic acid methyl ester not present in isomers of phthalate were extracted and summed for each sample. A linear regression model was fit between the base peak sums and the TIC areas of the pure docosanoic acid methyl ester peaks using Matlab. Outliers (*n* = 4) were removed based on calculated Cook's distances at a cutoff of 4/24. A Shapiro-Wilks test of the distribution of base peak sums indicated a normal distribution (*p* = 0.989, *W* = 0.988) and the regression model determined a strong linear fit between base peak sums and TIC areas (*R*^2^ = 0.986). Adjusted TIC areas for all PLFA samples in our dataset were subsequently calculated via the extraction and summation of the external standard base peaks and input into the aforementioned linear regression model. Once adjusted TIC areas were obtained for PLFA samples, the FAME concentrations were then calculated by linear proportion as described above.

### Statistical analysis

All extractions were carried out in triplicates, except the mBD + Phos treatments which were done in duplicates. The equivalent concentration of the amended lipids were subtracted from the samples and the internal standard and external standards were not considered in the yield calculations. Differences in PLFA and DGFA yield, diversity, and DGFA/PLFA ratios between treatment methods were analyzed using one-way Analysis of Variance (ANOVA) tests followed by Tukey HSD *post-hoc* tests in JMP Pro version 12.2.0 (SAS Institute, Cary, North Carolina). Analysis of Similarity (ANOSIM) test was also done for PLFA and DGFA datasets (α = 0.05). Significant differences are reported at α = 0.05 level. Non-metric multidimensional scaling (NMDS) analysis was conducted in R statistical software version 3.2.4 using the “stats” version 2.15.3 and “vegan” version 2.3-5 (Oksanen et al., 2016) packages. Specifically, Bray-Curtis distances were calculated from absolute FAME concentrations (pmol). The resulting distance matrices were used to calculate NMDS plots. One mBD + Phos sample was removed from the PLFA and DGFA NMDS analyses as an outlier. A second Folch sample was withdrawn from the DGFA NMDS analysis because the profiles contained only two saturated FAMEs. Vectors representing the correlation (*p* < 0.05) between samples and FAME classes were plotted to discern which types of FAMEs were driving the differences between samples. The relative abundances of FAME classes for PLFA and DGFA samples were regressed (α = 0.05, permutations = 999) against Bray-Curtis distances using the envfit function in the vegan package. The resulting arrow vectors were overlaid on the NMDS plot from the origin and represent the correlation of FAME class abundances to ordinated samples. The aim of the NMDS was to describe as closely as possible any clustering patterns based on observed FAMEs classes.

## Results

Lipid biomarkers from all extraction treatments for PL- and DG-FAMEs in mol% and pmol/g are shown in Tables 2, 3, respectively. Selected extract ion chromatograms (EIC; for m/z 74) are presented as Supplementary Information (Figures S1–S5).

**Table 2:** Molar percentages of PL-FAME, yield in pmol/g, and number of detected PL-FAME biomarkers recovered from the different extraction treatment methods.

**Extraction Type**	**Pho**	**Pho**	**Cit**	**Cit**	**Cit**	**Mg**	**Mg**	**Mg**	**POPC**	**POPC**	**POPC**	***E.coli***	***E.coli***	***E. coli***	**Folc**	**Folc**	**Folc**	**MAE**	**MAE**	**MAE**
**Sample ID**	**RT02-PL**	**RT01R-PL**	**RT07-PL**	**RT08-PL**	**RT09-PL**	**RT10-PL**	**RT11-PL**	**RT12-PL**	**RT13-PL**	**RT14-PL**	**RT15-PL**	**RT16-PL**	**RT17-PL**	**RT18-PL**	**RT19-PL**	**RT20-PL**	**RT21-PL**	**RT22-PL**	**RT23-PL**	**RT24-PL**
**PLFA**																					
C11:0	0.00	0.00	0.29	0.00	0.46	0.00	0.00	0.28	0.24	0.85	0.29	0.00	0.00	0.00	0.42	0.32	0.39	0.39	0.30	0.37
C12:0	0.00	0.00	0.00	0.00	0.00	0.00	0.00	0.00	0.00	0.00	0.00	0.00	0.00	0.00	0.11	0.00	0.00	0.11	0.10	0.00
C13:0	0.00	0.00	0.00	0.00	0.00	0.00	0.00	0.00	0.00	0.00	0.00	0.00	0.00	0.00	0.00	0.00	0.00	0.11	0.00	0.00
C12:0 2-OH	0.00	0.00	0.00	0.00	0.00	0.00	0.00	0.00	0.00	0.00	0.00	0.00	0.00	0.00	0.23	0.00	0.00	0.00	0.00	0.00
C14:0	0.00	0.00	1.35	0.98	2.33	0.00	0.00	0.00	4.29	5.21	5.53	0.00	0.00	0.00	2.28	1.18	0.00	3.70	2.96	2.66
C15:0	0.00	0.00	0.09	0.13	0.23	0.00	0.00	0.00	0.73	0.63	0.00	0.00	0.00	0.00	0.25	0.76	0.98	1.23	3.12	1.30
C16:1w9t	0.00	0.00	0.00	0.00	0.00	0.00	0.00	0.00	0.00	0.00	0.00	0.00	0.00	0.00	1.28	0.00	0.00	0.00	0.00	0.00
C16:1w9c	0.00	0.00	1.13	1.21	1.83	0.47	0.00	0.91	1.78	1.67	2.07	0.48	0.00	0.00	0.00	0.98	0.00	0.00	2.60	1.53
C16:0	29.95	23.47	36.27	37.78	34.84	31.07	29.60	28.68	31.52	33.66	33.50	27.72	27.91	24.84	30.18	23.55	31.29	34.26	32.99	36.12
iso-C17:0	0.00	0.00	0.00	0.00	0.00	1.22	0.00	0.00	0.00	0.00	0.00	0.00	0.00	0.00	0.00	0.00	0.00	0.00	0.00	0.00
12-cyclo-C18:1	0.00	0.00	0.00	0.00	0.00	0.00	0.00	0.50	0.00	0.00	0.00	0.00	0.00	0.00	0.00	0.00	0.00	0.00	0.00	0.00
anteiso-C17:0	0.00	0.00	0.00	0.31	0.80	0.00	0.00	0.00	1.39	0.98	2.06	0.00	0.00	0.00	0.00	0.51	0.86	0.41	0.00	0.00
C17:0	1.24	0.00	0.71	0.89	0.89	0.87	2.38	0.99	1.56	1.09	1.61	0.97	2.03	1.35	0.98	0.91	1.36	1.16	4.83	1.67
10OH-C18:2	0.00	0.00	0.00	0.00	0.00	0.00	0.00	0.44	0.35	0.00	0.00	0.00	0.00	0.00	0.00	0.00	0.41	0.00	0.00	0.00
C18:1 5-EP	1.19	0.00	0.00	0.00	0.00	0.00	0.00	0.00	0.00	0.00	0.00	0.00	0.00	0.00	0.00	0.00	1.24	1.12	0.00	0.00
C18:2ω6	0.00	0.00	1.03	0.00	0.96	0.00	0.00	0.00	1.46	1.35	1.42	0.00	0.00	0.00	0.00	23.05	0.00	0.00	0.00	0.00
C18:1ω9t	0.00	36.33	29.96	30.31	27.27	31.67	30.52	29.71	25.87	26.48	26.34	32.66	29.79	34.90	28.22	18.03	1.27	0.74	23.06	27.87
C18:1ω7c	0.00	6.22	6.21	5.94	6.09	5.29	6.44	7.09	6.40	5.93	6.03	6.98	7.37	6.25	5.55	5.11	0.62	0.38	8.82	6.07
C18:0	18.84	22.75	13.37	14.27	12.23	18.31	18.67	15.00	11.71	11.96	11.41	16.71	17.17	20.62	15.88	11.06	15.54	14.32	13.36	14.16
9-Epoxy-C18:1t	3.30	2.67	2.44	2.18	2.65	2.31	3.61	4.29	2.71	2.79	2.32	6.45	6.63	2.74	3.62	4.29	6.31	3.32	2.01	2.16
C18:2w8	0.00	0.00	0.00	0.00	0.00	0.00	0.00	0.00	0.00	0.00	0.00	0.00	0.00	0.00	0.00	0.00	2.01	1.34	0.00	0.00
9-Epoxy-C18:1c	30.57	1.87	1.39	1.37	1.81	2.07	1.96	2.30	1.73	1.35	1.78	2.13	2.79	2.29	2.03	1.68	18.42	20.19	2.25	1.66
C18:0 10-OX	4.94	4.60	3.65	3.41	4.06	4.22	4.56	5.10	4.03	3.82	3.78	4.08	4.76	3.70	5.30	3.56	4.93	5.03	3.59	4.43
C20:1ω9	0.00	0.00	0.51	0.00	0.74	0.00	0.00	0.39	0.39	0.00	0.00	0.36	0.00	0.00	0.00	0.00	0.00	0.00	0.00	0.00
9,10-OH-C18:0	0.00	0.00	0.00	0.00	0.00	0.00	0.00	0.00	0.00	0.00	0.00	0.00	0.00	0.00	0.00	0.00	0.40	0.51	0.00	0.00
10-OH-C18:1	0.00	0.00	0.00	0.00	0.44	0.00	0.00	0.00	0.53	0.00	0.00	0.00	0.00	0.00	0.00	0.29	0.00	0.00	0.00	0.00
C20:0	1.76	2.08	1.40	1.22	1.66	2.52	2.27	2.16	1.28	1.13	1.25	1.46	0.00	2.27	3.20	2.59	2.57	2.27	0.00	0.00
9,10-Cyclo-C18:2	0.00	0.00	0.00	0.00	0.00	0.00	0.00	0.00	0.00	0.00	0.00	0.00	0.00	0.00	0.00	0.41	0.53	0.00	0.00	0.00
C21:0	0.00	0.00	0.19	0.00	0.00	0.00	0.00	1.24	0.60	0.33	0.61	0.00	1.54	1.04	0.46	0.86	0.00	0.00	0.00	0.00
9,10-Chloro-C18:0	2.19	0.00	0.00	0.00	0.00	0.00	0.00	0.00	0.00	0.00	0.00	0.00	0.00	0.00	0.00	0.00	2.66	2.54	0.00	0.00
9,10-Chloro-C18:0	6.03	0.00	0.00	0.00	0.00	0.00	0.00	0.00	0.32	0.38	0.00	0.00	0.00	0.00	0.00	0.00	6.83	5.99	0.00	0.00
C23:0	0.00	0.00	0.00	0.00	0.12	0.00	0.00	0.00	0.34	0.00	0.00	0.00	0.00	0.00	0.00	0.30	0.00	0.00	0.00	0.00
C24:0	0.00	0.00	0.00	0.00	0.58	0.00	0.00	0.91	0.75	0.39	0.00	0.00	0.00	0.00	0.00	0.56	1.36	0.87	0.00	0.00
PLFA Yield	85.0	48.30	240.8	211.6	704.2	90.82	108.9	341.9	376.3	350.2	474.1	83.93	140.4	39.69	199.1	464.7	310.1	350.2	309.1	203.6
Number of PL-FAMES	10	8	15	13	19	11	9	16	22	18	15	11	9	10	16	20	20	21	13	12
**FUNCTIONAL GROUP TOTALS**																					
N-Sats	51.79	48.30	53.38	55.28	52.89	52.76	52.91	48.99	52.78	54.41	53.90	46.86	48.66	50.12	53.35	41.76	53.11	58.04	57.36	55.91
M-Unsats	0.00	42.55	37.81	37.46	35.93	37.42	36.96	38.10	34.44	34.08	34.44	40.48	37.16	41.15	35.05	24.13	1.89	1.12	34.48	35.47
Epoxy-	35.05	4.55	3.84	3.55	4.46	4.37	5.57	6.59	4.44	4.14	4.10	8.58	9.41	5.03	5.65	5.97	25.98	24.63	4.26	3.82
Hydroxy-	0.00	0.00	0.00	0.00	0.44	0.00	0.00	0.44	0.89	0.00	0.00	0.00	0.00	0.00	0.23	0.29	0.81	0.51	0.00	0.00
Keto-	4.94	4.60	3.94	3.41	4.52	4.22	4.56	5.38	4.27	4.67	4.07	4.08	4.76	3.70	5.72	3.88	5.32	5.41	3.89	4.81
T-Branche	0.00	0.00	0.00	0.31	0.80	1.22	0.00	0.00	1.39	0.98	2.06	0.00	0.00	0.00	0.00	0.51	0.86	0.41	0.00	0.00
PolyUnsats	0.00	0.00	1.03	0.00	0.96	0.00	0.00	0.00	1.46	1.35	1.42	0.00	0.00	0.00	0.00	23.05	2.01	1.34	0.00	0.00
Cyclopropy	0.00	0.00	0.00	0.00	0.00	0.00	0.00	0.50	0.00	0.00	0.00	0.00	0.00	0.00	0.00	0.41	0.53	0.00	0.00	0.00

**Table 3:** Molar percentages of DG-FAME, yield in pmol/g, and number of detected DG-FAME biomarkers recovered from the different extraction treatment methods.

**Extraction Type**	**Phos**	**Phos**	**Cit**	**Cit**	**Cit**	**Mg2+**	**Mg2+**	**Mg2+**	**POPC**	**POPC**	**POPC**	***E. coli***	***E. coli***	***E. coli***	**Folc**	**Folc**	**MAE**	**MAE**	**MAE**
**Sample ID**	**RT02_DG**	**RT01R_DG**	**RT07_DG**	**RT08_DG**	**RT09_DG**	**RT10DG**	**RT11_DG**	**RT12_DG**	**RT13_DG**	**RT14_DG**	**RT15_DG**	**RT16_DG**	**RT17_DG**	**RT18_DG**	**RT19_DG**	**RT21_DG**	**RT2_DG**	**RT2_DG**	**RT24_DG**
**DGFA**																			
C11:0	0.00	0.00	0.00	0.00	0.00	0.00	0.00	0.00	0.00	0.00	0.00	0.00	0.00	0.00	0.00	0.00	0.06	0.00	0.03
C12:0	0.17	0.00	0.15	0.00	0.00	0.00	0.12	0.47	0.29	0.12	0.11	0.07	0.00	0.00	0.12	0.00	0.50	0.08	0.36
C13:0	1.39	0.10	0.00	0.00	0.00	0.00	0.70	0.20	0.89	0.22	0.29	0.35	0.00	0.00	0.57	0.00	1.14	0.43	1.50
iso-C14:0	0.81	0.21	0.00	0.00	0.00	0.00	0.00	0.00	0.45	0.21	0.25	0.28	0.00	0.00	0.50	0.00	0.47	0.34	0.77
C14:0	3.85	3.76	1.51	4.39	2.42	3.12	3.43	4.84	3.30	2.16	2.50	2.63	0.56	0.61	2.94	1.56	3.74	3.64	4.57
C15:0	4.37	0.00	4.89	5.39	5.12	3.57	6.75	0.00	4.97	4.25	5.04	4.65	1.99	1.52	4.99	4.56	6.28	5.99	7.52
C16:1w14t	5.64	4.25	5.66	9.40	6.71	11.58	1.20	6.90	4.63	4.87	4.56	4.49	2.96	2.62	5.52	2.51	3.12	0.00	0.00
C16:0	25.19	26.98	13.86	19.44	18.53	21.49	29.20	24.98	20.57	19.46	21.02	25.03	29.44	29.94	21.22	40.56	29.92	25.72	22.42
anteiso-C17:0	7.54	6.76	7.19	14.15	10.08	0.00	0.00	0.00	5.06	5.71	6.36	4.57	3.24	3.33	4.69	4.52	5.40	5.82	5.15
C17:0	7.26	7.25	7.26	9.71	12.75	4.85	6.14	12.17	4.63	6.37	4.58	5.41	3.09	3.35	5.78	4.24	5.51	6.41	5.26
C18:2w6	3.61	6.31	0.00	0.00	0.00	0.00	0.00	0.00	3.30	3.53	3.39	2.72	2.22	2.46	0.00	0.00	0.00	0.00	0.00
C18:3w3	0.00	0.00	0.00	0.00	0.00	0.00	0.00	0.00	1.91	2.57	0.00	0.00	0.00	0.00	0.00	0.00	3.39	0.00	2.57
C18:1w9c	0.00	18.25	12.56	15.45	13.70	21.84	22.74	17.07	18.09	19.12	19.00	20.37	28.53	27.23	22.07	0.00	4.19	0.00	18.52
C18:1w9t	0.00	8.45	8.77	8.94	9.11	11.15	8.07	7.75	7.51	7.52	7.02	6.75	5.75	5.86	7.50	0.00	5.52	23.69	8.45
4OH-18:0	0.00	0.00	0.00	0.00	0.00	0.00	0.00	0.00	1.69	2.22	4.31	0.00	0.00	0.00	0.00	0.00	1.38	0.00	0.00
C18:0	17.01	13.01	12.00	10.21	13.80	13.82	15.06	16.69	13.37	14.41	13.00	13.73	18.06	18.20	13.89	22.31	18.85	17.70	13.47
cyC19:0	5.82	4.35	5.27	2.92	7.78	8.59	1.27	7.27	3.82	4.16	3.72	3.55	1.15	1.68	4.59	2.91	4.07	5.22	4.76
6-epoxy-C18:1t	4.46	0.00	4.48	0.00	0.00	0.00	4.84	0.00	2.59	2.72	4.48	2.23	1.56	1.76	3.08	2.86	3.38	4.17	3.55
9-epoxy-C18:1c	6.79	0.00	0.00	0.00	0.00	0.00	0.00	1.66	0.00	0.00	0.00	0.00	0.00	0.00	0.00	1.79	2.14	0.00	0.00
C20:1w9t	0.00	0.00	0.00	0.00	0.00	0.00	0.00	0.00	2.60	0.00	0.00	2.56	1.36	1.00	0.00	0.00	0.00	0.00	0.00
12-cyclo-C18:1t	1.84	0.00	0.00	0.00	0.00	0.00	0.00	0.00	0.00	0.00	0.00	0.00	0.00	0.00	0.76	2.08	0.00	0.00	0.00
9,10-chloro-C18:0	3.00	0.00	0.00	0.00	0.00	0.00	0.00	0.00	0.00	0.00	0.00	0.20	0.00	0.00	0.59	9.28	0.00	0.00	0.00
C23:0	0.55	0.09	16.42	0.00	0.00	0.00	0.12	0.00	0.00	0.08	0.12	0.12	0.09	0.16	0.45	0.00	0.10	0.34	0.31
DGFA Yield (pmol/g)	1356	1959	905	815	811	257	1418	389	3040	2479	3953	2528	752	1108	2921	258	2786	3155	3229
Number of DG-FAMEs	18	14	12	10	10	9	14	11	20	19	18	19	14	15	18	13	20	14	17
DGFA:PLFA Ratio	16	41	4	4	1	3	13	1	8	7	8	30	5	28	15	1	8	10	16
**FUNCTIONAL GROUP TOTALS**																			
N-Sats	60.50	51.43	46.85	61.88	59.35	52.28	53.23	54.05	50.70	74.05	49.14	56.07	52.62	48.03	47.31	46.94	66.33	60.92	55.93
Mo-unsats	5.64	30.94	44.57	32.01	31.72	34.16	38.61	36.72	35.09	2.51	33.79	27.00	29.52	32.83	31.51	30.58	12.83	23.69	26.97
Epoxy-	11.24	0.00	0.00	4.84	1.66	2.23	1.56	1.76	3.08	4.66	0.00	4.48	0.00	2.59	2.72	4.48	5.52	4.17	3.55
Hydroxy-	0.00	0.00	0.00	0.00	0.00	0.00	0.00	0.00	0.00	0.00	0.00	0.00	0.00	1.69	2.22	4.31	1.38	0.00	0.00
T-Branched	8.34	6.97	0.00	0.00	0.00	4.86	3.24	3.33	5.19	4.52	14.15	7.19	10.08	5.51	5.92	6.61	5.87	6.15	5.92
PolyUnsats	3.61	6.31	0.00	0.00	0.00	2.72	2.22	2.46	0.00	0.00	0.00	0.00	0.00	5.20	6.10	3.39	3.39	0.00	2.57
Cyclopropy	7.65	4.35	8.59	1.27	7.27	3.55	1.15	1.68	5.35	4.98	2.92	5.27	7.78	3.82	4.16	3.72	4.07	5.22	4.76
Chloro-	3.00	0.00	0.00	0.00	0.00	0.20	0.00	0.00	0.59	9.28	0.00	0.00	0.00	0.00	0.00	0.00	0.00	0.00	0.00

Phos = mBD + Phos, Cit = mBD + Cit, Mg2+ = mBD + Phos + Mg2+, POPC = mBD + Phos + POPC, E coli = mBD + Phos + E coli, Folch = Folch, MAE = Microwave Assisted Extraction

*N-Sats = normal saturates, Mo-unsats = monounsaturates, T-Branched = terminally branched, polyunsats = polyunsaturates*,

*DGFA/PLFA = Diglyceride fatty acid pmol/g to polar lipid fatty acid pmol/g ratio*.

### FAME yields

**Figure 2:**
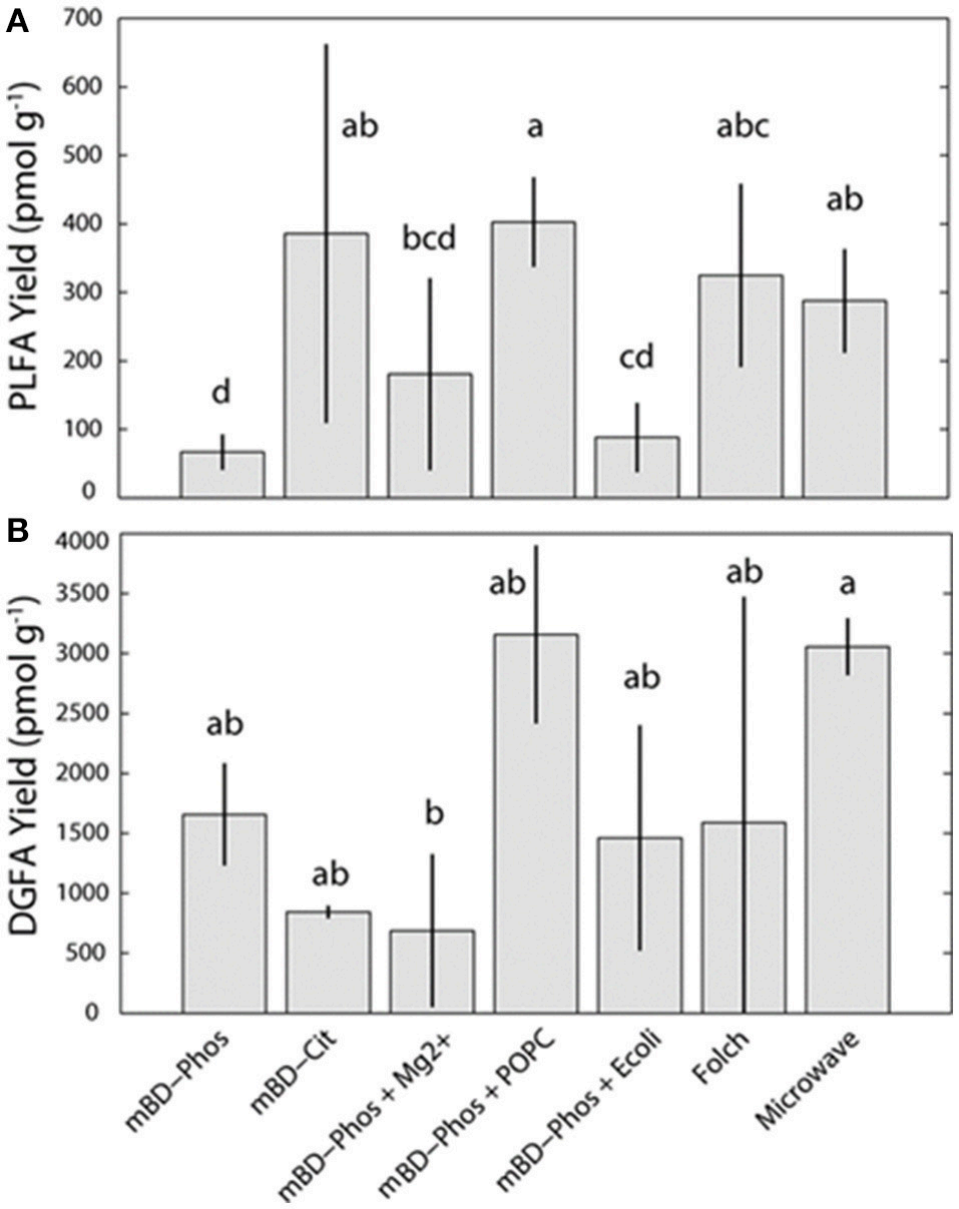
Average PL **(A)** and DG-FAMEs **(B)** for each extraction treatment method (*n* = 7). Error bars represent the standard deviation between replicates. Shared letters indicate no significant differences in mean concentration, based on ANOVA and Tukey-HSD tests (α = 0.05), are plotted above each bar.

The average PL-FAME yields ranged from 67 to 400 pmol/g (Figure 2A). Average yields for the mBD + Cit, mBD + Phos + POPC, and MAE were similar among each other and were significantly different from the mBD + Phos, mBD + Phos + Mg^2+^, and mBD + Phos + *E. coli* treatments (ANOVA with Tukey HSD *post-hoc* test, α = 0.05). The average DG-FAME yields ranged from 600 to 3,000 pmol/g (Figure 2B). Except for the MAE and mBD + Phos + Mg^2+^ treatment methods, yields for the DG-FAMEs extraction treatment methods did not show any statistical difference (ANOVA with Tukey HSD *post-hoc* test, α = 0.05). The mBD + Phos + POPC outperformed the standard un-amended mBD + Phos extraction with a ~6 fold increase in PLFA yield and ~5 fold increase in DGFA yield compared to the mBD + Cit. (Figures 2A,B).

### Fame structural classes and DGFA/PLFA response

**Figure 3:**
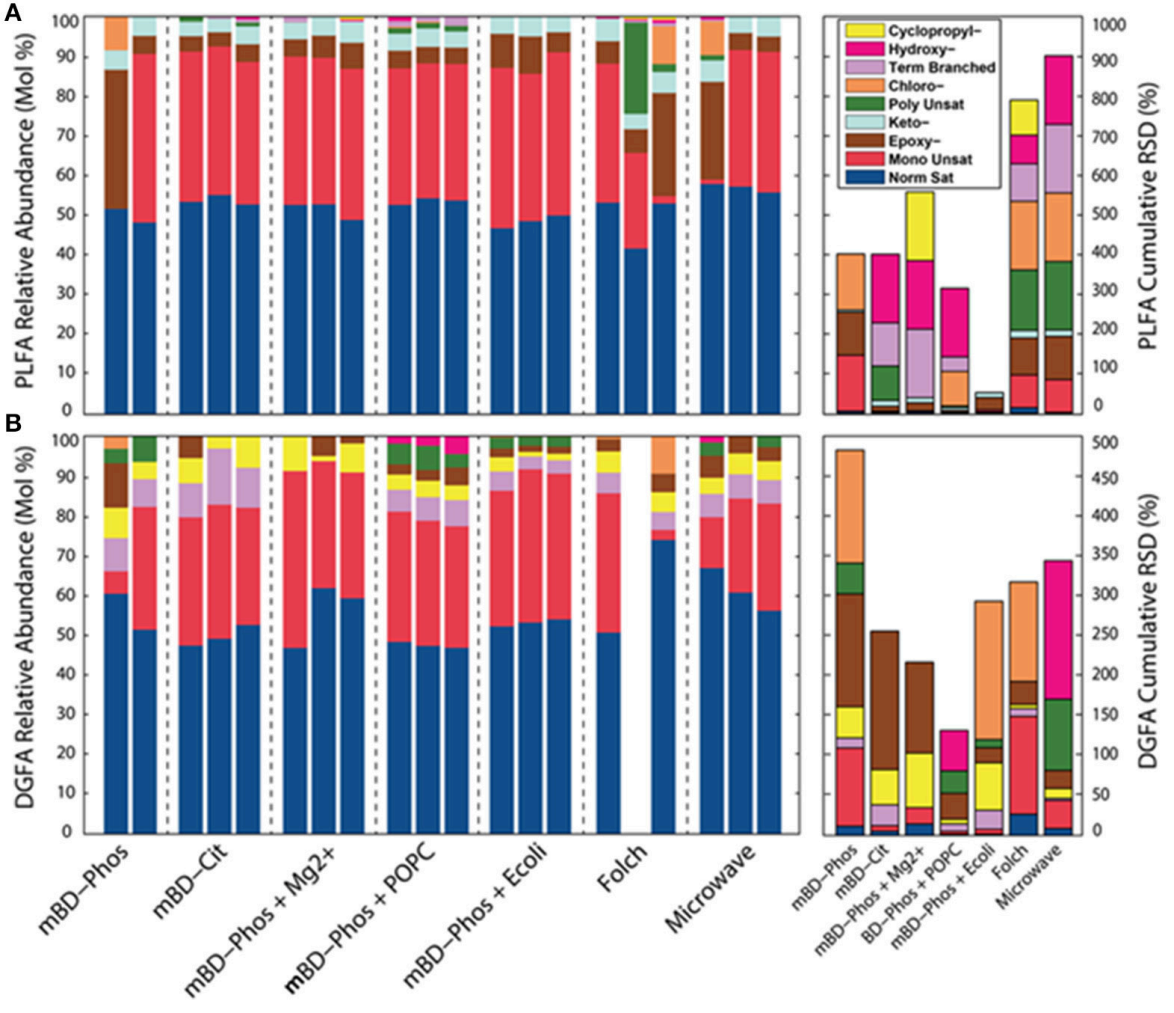
Relative abundances of PL (*n* = 20, **A**) and DG-FAME (*n* = 19, **B**) profiles based on the classes of each sample across all extraction treatments (*n* = 7), and RSD measurements for PLFA and DG-FAME classes for each treatment.

**Figure 4:**
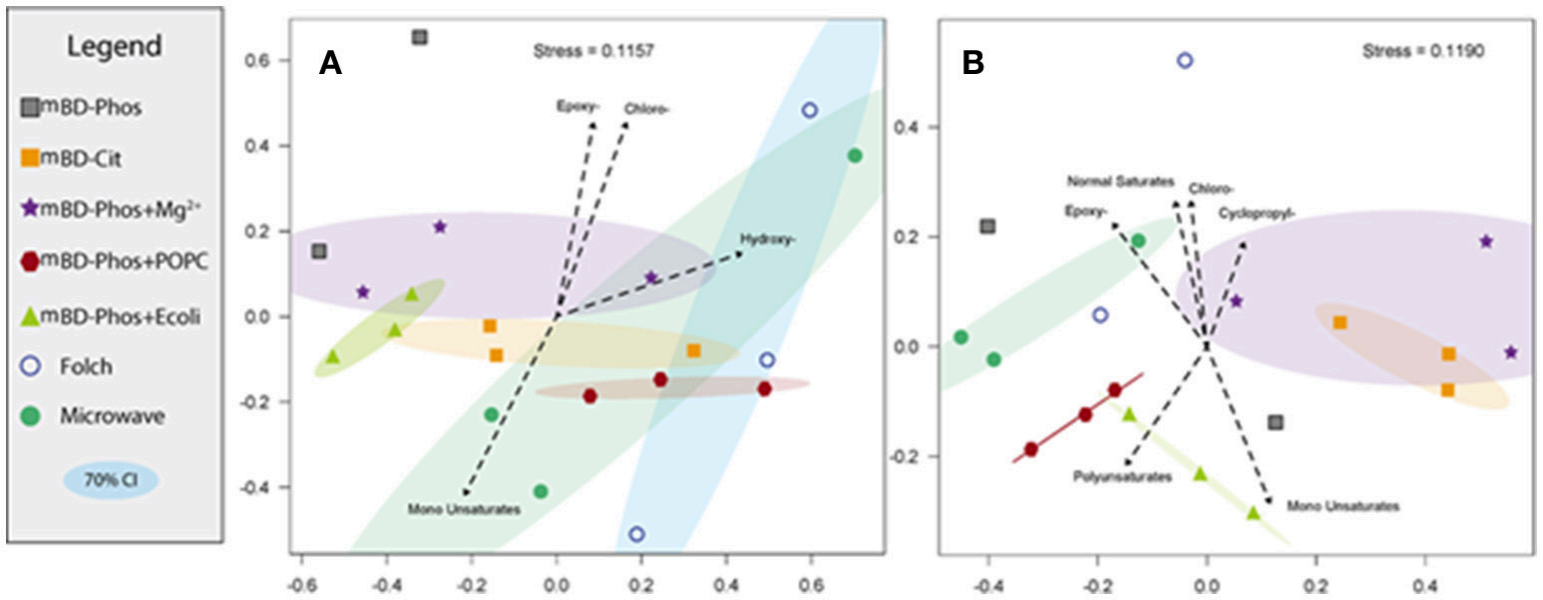
Non-metric Multi-Dimensional Scaling (NMDS) of experimental samples for both PL **(A)** and DG-FAMEs **(B)**. Vectors representing significant (α = 0.05) correlations of FAME relative abundance were added to reveal significant drivers between groupings. Confidence intervals (70%) for each treatment grouping were also plotted.

**Figure 5:**
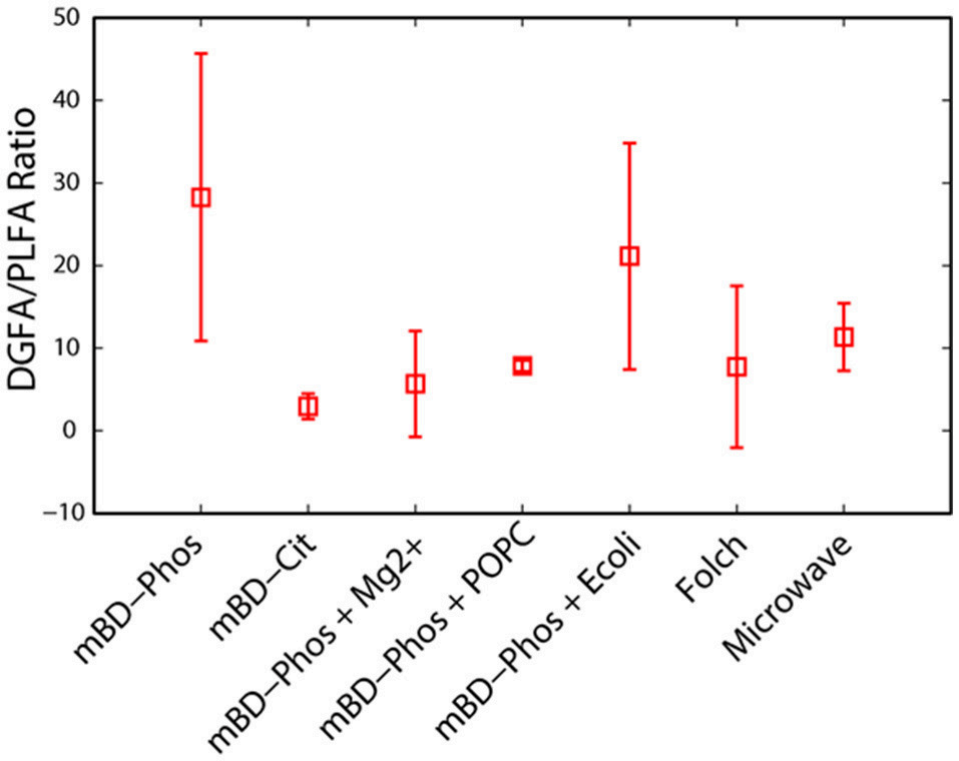
A comparison of the ratio of DG to PL-FAMEs across extraction treatment methods (*n* = 7).

The low abundance FAME classes (<10% relative abundance) exhibited the most variability between treatment methods while the high abundance FAME classes (>10% relative abundance) were consistent both within and across treatments (Figures 3A,B). The mBD + Phos + POPC samples exhibited high reproducibility and least variability for the low abundant FAME classes for both PLFA and DGFA. Other treatment methods like the mBD + Phos + *E. coli* and MAE also had relatively less variability for the low abundance FAME classes for the DGFA only. NMDS analysis showed samples clustering together based on extraction treatment type (*p* = 0.003, Figure 4). The average DGFA:PLFA ratio between treatments ranged from ~2 to 27 (Figure 5).

## Discussion

### Influence of extraction treatments on total lipid yield (microbial biomass)

#### Amended vs. un-amended standard Bligh and Dyer procedure

The primary aim of our study was to determine the efficiency of specific lipid extraction treatments and establish an optimized extraction protocol for shale core samples. When the standard Bligh and Dyer method using a phosphate buffer (mBD + Phos) was amended with an intact phospholipid (mBD + Phos + POPC), the shale samples yielded more lipid biomarkers and better reproducibility for both the PLFA and DGFA (Figures 2A,B). Black shales are generally characterized by high amounts of clay minerals, salinity, carbonates, organic matter, and other minerals like quartz and feldspars (Shaw and Weaver, 1965; Boles and Franks, 1979; Aplin and Macquaker, 2011; Chermak and Schreiber, 2014). Studies have shown how interactions between fatty acids, clay minerals, (Meyers and Quinn, 1973; Morris and Calvert, 1975; Boles and Franks, 1979; Lahann and Campbell, 1980; Aplin and Macquaker, 2011; Chermak and Schreiber, 2014), and carbonates (Zullig and Morse, 1988; Thomas et al., 1993) could impede efficient lipid extraction. Such mineral-lipid interactions have been shown to depend on a combination of the isoelectric point of the minerals, physical adsorption, electrostatic, van der Waals, and chemical bonding (Stevens et al., 2009; Oleson et al., 2010; Sahai et al., 2017). Amphipathic compounds (11-mercaptoundecanoic acid, MUA and 1-dodecanethoil, DDT) have been used to minimize the interference of such mineral-lipid interactions through self-assembly of lipid molecules in solution (Lee et al., 2014). In the presence of these compounds, the lipid molecules aggregated, while in their absence the lipid molecules remained un-aggregated in solution. These observations were attributed to hydrophobic interactions, dynamic rearrangement of the biochemical compounds on the particle surfaces, and short ranged electrostatic forces on the particle surfaces.

Accordingly, surface charge adsorption of intact phospholipid ditridecanoylphosphocholine (DTPC) and 1-palmitoyl-2-oleoyl-sn-glycerol-3-phosphocholine (POPC) have been observed on common minerals present in shale (Kalb et al., 1992; Xu et al., 2009). We therefore propose that a similar interaction between the hydrophobic and hydrophilic segments of the POPC and the mineral matrix could be responsible for the aggregation or self–assembly of the lipid molecules in solution, enhancing their efficient solvent recovery. Sahai et al. (2017), used a model and suggested that the adsorption of the added lipid molecules on the mineral surface acted as a template for the assembly of more lipid molecules in solution. As a result, we suggest that the intact POPC in our treatment was the catalyst for mediating the lipid bilayer assembly, decreasing the microbial lipid adsorption in solution and thus increasing potential for solvent recovery. The re-extraction step might have also increased the effectiveness of the added lipid (POPC) performance, by increasing the available reactive surface areas for the interactions between the intact POPC, the shale matrix, and the shale bound lipids. Other researchers have observed 5–10% (Wu et al., 2009) and ~20% (Papadopoulou et al., 2011) increase in lipid recovery with re-extractions. In this case, both processes (i.e., POPC addition and re-extraction steps) could potentially have resulted in significant amounts of lipid recovery and better reproducibility. It is possible that a similar interaction could also occur between the internal standard (1, 2-dinonadecanoyl-sn-glycero-3-phosphocholine) and the shale matrix of the samples which could also lead to the improvement of yields. However, this effect would be applicable to all the extraction treatments, since the same amount of internal standard was added to the treatments. It is also important to note due to the highly heterogeneous nature of the deep subsurface system and the limited number of replicates (*n* = 3) in these samples, we cannot say with certainty if the reproducibility in the POPC treatments can be repeatable across large number of replicates.

The *E. coli* and Mg^2+^ amendments were not effective in allowing more lipid recovery from the shale samples as expected (Figures 2A,B). This was unexpected because the *E. coli* was calculated to provide a similar concentration of exogenous additive similar to the POPC amendment based on the conversion factor in Kieft et al. (1994). The addition of 1,200 ppm Mg^2+^ was also intended to reduce the adsorption of fatty acids (Lahann and Campbell, 1980), thereby increasing the extraction efficiency. Though the actual reason for the discrepant performance is unknown, it is probable that Mg^2+^ ions and microbial cells from the *E. coli* may not be suitable for lipid recovery from complex matrices like shale, but could be suitable to improve recovery from samples of different matrices. Differential performance of extraction solutions have been observed with lipid recovery. For example, optimized extraction solution performance has been reported in samples of high mineral and salt content while samples of low mineral content did not show similar optimized recovery (Christie, 1993; Gómez-Brandón et al., 2008). The authors suggested that the buffer:reagents were effective in interacting with the high mineral and salt content of the matrices thereby improving yields, while such similar interactions were absent in the samples of simpler matrices. Frostegard et al. (1991), also examined the efficiency of extraction treatments in samples of various matrices and found that some treatments were effective in samples of high organic matter content but less effective in samples of low organic matter content. These disparities in yield among treatment methods and matrices is a confirmation that some solvent:reagent:buffer combinations might be effective in lipid recovery from samples of particular matrix properties, but not effective in samples of different matrices. While we could not establish a clear explanation for the poor performance, it is apparent that the Mg^2+^ and *E. coli* amendment interfered with the lipid recovery for the PLFA and further research will be needed to explain this phenomena.

#### Modified Folch and microwave assisted extraction procedure

While we showed an improvement in lipid recovery between the POPC amended treatment and the un-amended Bligh and Dyer phosphate buffered treatment, we did not observe any significant difference in yield between the POPC treatment, Folch, mBD + Cit, and MAE methods (Figures 2A,B). Previous comparisons between the Bligh and Dyer phosphate buffered method (mBD + Phos), the Folch, and MAE methods from samples of different matrices (manure, compost, vemicompost, and soil) showed that the Folch method outperformed the un-amended Bligh and Dyer (mBD + Phos) which in turn outperformed the MAE method (Gómez-Brandón et al., 2008, 2010). In contrast, our observations show that both Folch and MAE outperformed the un-amended mBD + Phos but not the POPC amended Bligh and Dyer (mBD + Phos + POPC) treatment (Figures 2A,B). This observed improved performance of the Bligh and Dyer amended treatment compared to Folch and MAE could be directly associated with the addition of the POPC amendment. Hence, these observations corroborate the suggestion that the addition of the exogenous POPC in the extraction solvent may have improved the performance of the traditional Bligh and Dyer phosphate buffered method in lipid recovery.

Additionally, our results also show an optimized performance for the MAE treatment. Gómez-Brandón et al. (2008, 2010), reported that the MAE method had the lowest performance in lipid recovery from samples of both high and low organic matter matrices when compared to the standard Bligh and Dyer (mBD + Phos), and Folch methods. Contrastingly, our results indicate that the MAE outperformed the mBD + Phos and performed equally well with the Folch method (Figures 2A,B). Since the response of the MAE was higher, it is possible that this improvement could be due to the modifications made on the extraction solvents. Previous MAE extraction treatments utilized a combination of hexane:acetone as extraction solvents (Lopez-Avila et al., 1995; Gómez-Brandón et al., 2008, 2010) while our extraction solvents were modified to chloroform:methanol. Although chloroform:methanol has generally been considered more effective extraction reagents for lipids from environmental samples (Ewald et al., 1998; Renaud et al., 1999), their lethality and environmental unfriendliness is why most studies prefer other reagents. The effectiveness of solvents in deep subsurface samples is particularly essential as most microbes in deep subsurface aquifer settings are always attached to sediment matrix (Franzmann et al., 1996; Murphy et al., 1997; Ginn et al., 1998), and will require appropriate reagents to be efficiently released (Thomas et al., 1993). We argue that in addition to optimizing lipid recovery between the amended and un-amended phosphate buffered treatment method, our solvent modification was also responsible for the improved performance of the MAE treatment method.

In a similar fashion, the buffer type in the extraction solution also influences lipid recovery. Gómez-Brandón et al. (2010) suggested that a suitable buffer during extraction could prevent loss of lipids into the aqueous phase of the extraction mixture by reducing ionization effects. Other researchers have also proposed that interactions between organic content of samples and pH of the buffer could also affect lipid yield (Frostegard et al., 1991; Nielsen and Petersen, 2000). Using soil samples of high organic matter content, Frostegard et al. (1991) reported higher lipid recovery with citrate buffer (pH 4) as opposed to the standard phosphate buffer (pH 7.4). Comparably, Nielsen and Petersen (2000) also observed an increase in lipid recovery with citrate buffer rather than phosphate buffer. The authors suggested that the acid nature of the citrate buffer reduced organic matter interference with shale bound lipids, thereby preventing the adsorption of microbial lipids on the matrix. Citrate has also been reported to be involved in chelating cations and metals (Glusker, 1980) and as such could be responsible for the interaction between the organic matter and the microbial lipids. These findings, therefore emphasizes the significance of citrate chelation in samples of high organic matter content like black shales. More so, Nielsen and Petersen (2000) proposed that organic matter interference could lead to about 20% reduction in lipid recovery. Consequently, the relatively high lipid recovery for the mBD + Cit samples compared to the standard un-amended mBD + Phos treatments (Figures 2A,B) could be explained by the effectiveness of the citrate buffer in improving lipid recovery in samples of high organic matter content by minimizing ionic adsorption.

### Influence of extraction treatment on lipid reproducibility

We observed differential performance especially with the unique FAMEs of low abundance (<10% relative abundance) across treatment methods, but not with the high abundant (<10% relative abundance) common FAMEs (Figures 3A,B). This high proportional variation both within and across treatment methods for the low abundant FAMEs was one of the primary objectives of our study. The extraction treatment with the ability to effectively recover these low abundant FAMEs for both the PLFA and DGFA was considered to be the most efficient procedure. This is because differential interaction between extraction solvents and samples of high organic and mineral content could increase the chances of obtaining highly variable results for lipid recovery. For example, Gómez-Brandón et al. (2010) observed higher proportional differences between the extraction methods for the organic rich samples (compost, vemicompost, and manure) while samples of less organic matter content (soil) showed less variations across treatment methods. Concomitantly, we believe that the physical (low porosity/permeability) and chemical (high mineral and organic content) properties of these samples are the underlying reason we see such large variations across replicates either through adsorption, sequestration, or interference leading to differential performance. The standard deviation by GC detection based on the external standard from the buffer control samples (*n* = 7) was 14.73%, implying that in addition to extraction treatment procedures, variations in GC detection could also influence the variability between treatments. It is also important to note that even in well mixed subsurface samples there could still be some variability. Studies have reported that even centimeter scale changes in depth could have predominant effects on microbial variability in the communities of deep subsurface samples (Brockman et al., 1992; Zhang et al., 1998). The authors reported that the microbial communities of deep subsurface were more isolated, existing in little niches or “islands.” Consequently, microbial examination may recover sulfate reducing bacteria (SRB) from one sample and not see it in a sample two cm away. It is therefore possible that when mixing those samples together, one may not get the community disperse. This could also partly explain the high error bars observed in the average yield of some of the extraction treatments (Figures 2A,B). These challenges further necessitate the continuous customized improvement of microbial lipid extraction procedures especially for samples with complicated matrices, such as deeply buried shales which could impede or bias findings related to microbial cell abundance and diversity.

The total number of FAME structural classes extracted using the intact POPC additive was higher and consistent within the triplicates for both the PLFA and DGFA compared to the mBD + Phos (Figures 3A,B). This finding led us to interpret that the PO PC amended treatment improved the effectiveness of the mBD + Phos method to obtain optimal microbial lipid diversity. Other treatment methods like the MAE and the *E. coli* amended treatment also recorded relatively good reproducibility for the DGFA only. Samples extracted with the Folch and MAE methods also had high total numbers of FAME structural classes, which aligned with our suggestion that the solvent modification was effective in improving recovery for the MAE method. However, considering the importance of establishing both the reproducibility and efficiency of microbial lipid biomarkers, the POPC still proved more suitable. For example, the Folch and MAE replicate samples failed to show repeatability among the FAMEs of low abundance. When we plotted vectors representing the correlation between samples and FAME classes, we found that no high abundance FAME was responsible for determining the differences between treatments. Rather, the low abundance FAMEs of individual samples within treatments were responsible for the increased within-treatment variation. Comparison by NMDS analysis (Figures 4A,B) showed samples from the same treatment methods with similar cluster patterns (*P* = 0.003). The replicates for the mBD + Phos + POPC, mBD + Phos + *E. coli*, and mBD + Cit samples were closest to each other with the smallest 70% confidence intervals. Extraction treatments with large 70% NMDS confidence intervals (Figures 4A,B) also had the highest cumulative RSDs for PL-and DG-FAME class relative abundances between the triplicates. Although some groupings had noticeable overlaps, the general trend remained the same.

### Influence of extraction treatments on DGFA/PLFA response

Besides determining the influence of extraction treatment methods on the lipid yields and reproducibility, our experimental design also allowed us to assess the variability of the interactions between the PLFA and DGFA across treatments. A DGFA–PLFA ratio provides a relative measure of nonviable to viable bacterial biomass (Kieft et al., 1994). A DGFA/PLFA ratio of 1 indicates equivalent amounts of viable and non-viable biomass. The average DGFA–PLFA ratio varied between treatment methods ranging from ~2 to 27 (Figure 5). Although most extraction treatments performed differently between the PLFA and DGFA, we did not observe any statistical difference between the treatment methods. However, the average yields for the DGFA were relatively higher than the PLFA across treatments methods (Figures 2A,B). We did not expect the yield for PLFA and DGFA biomarkers to be exactly the same since they both represent different kinds of lipid biomarkers (Kieft et al., 1994; Haldeman et al., 1994, 1995; White and Ringelberg, 1998; Fredrickson et al., 1997; Ringelberg et al., 1997). The DGFAs are more stable and less polar while the PLFAs are fragile and polar. Therefore, it is not surprising that we might see differences in PLFA and DGFA performances. The relatively higher yields in the DGFA across most extraction treatments could also be explained by the conversion of PLFA–DGFA during the concurrent breakdown of subsurface microbial cells during subsurface drilling and sampling (Haldeman et al., 1993, 1995). In addition, stressful environmental conditions associated with subsurface rocks could increase the likelihood of cell dead thus leading to higher DGFA concentrations as opposed PLFAs. More so, the fact that these samples were stored under room temperature conditions could have also favored the degradation of PLFA–DGFA, thus increasing the DGFA yield. Our objective was to choose the method that could perform well for both the PLFA and DGFA biomarker profiles.

## Conclusions

Our results showed that the choice of extraction treatment influenced the yield of the FAMEs. The lipid recovery efficiency of the Bligh and Dyer phosphate buffered method (mBD + Phos) was improved as a result of amendment with the intact phospholipid (POPC) for both PLFA and DGFA. The mBD + Phos + POPC treatment also exhibited higher recovery of unique lipids of low abundance for both PLFA and DGFA. When compared with previous performance to the Folch, mBD + Phos, and mBD + Cit, the MAE extraction was also improved by using chloroform:methanol as solvent extraction solution. The efficiency of the MAE was higher for the DGFA compared to the PLFA biomarkers. Higher lipid yield was observed for the citrate buffered Bligh and Dyer method compared to the standard un-amended Bligh and Dyer phosphate treatment. The Mg^2+^ and *E. coli* amendments did not prove to be efficient in the recovery of lipid biomarkers from the shale samples. Due to observed variations in performance of extraction treatments, we thus suggest that each extraction procedure should always be guided by both the sample matrix as well as the choice of targeted lipid biomarker. These methodological developments will thus provide better assessment of the microbial abundance of the deep surface as well as the role of environmental and energy applications on the deep subsurface microbial community.

## Author contributions

RT and PM were involved in the initial conception and design of the experiment. RA, RT, and SP were involved in subsequent experimental design, acquisition, and analysis of data. RA, RT, PM, and SS were involved in the data interpretation. RA, and RT were involved in drafting the manuscript. RA, RT, SP, PM, and SS were all involved in revising the manuscript for publication.

### Conflict of interest statement

The authors declare that the research was conducted in the absence of any commercial or financial relationships that could be construed as a potential conflict of interest.

## References

[B1] Aplin A. C. Macquaker J. H. S. (2011). Mudstone diversity: origin and implications for source, seal, and reservoir properties in petroleum systems. Am. Assoc. Petrol. Geol. Bull. 95, 2031–2059. 10.1306/03281110162

[B2] Batista A. Vetter W. Luckas B. (2001). Use of focused open vessel microwave-assisted extraction as prelude for the determination of the fatty acid profile of fish–a comparison with results obtained after liquid-liquid extraction according to Bligh and Dyer. Eur. Food Res. Technol. 212, 377–384. 10.1007/s002170000240

[B3] Biddle J. F. Lipp J. S. Lever M. A. Lloyd K. G. Sørensen K. B. Anderson R.. (2006). Heterotrophic Archaea dominate sedimentary subsurface ecosystems off Peru. Proc. Natl. Acad. Sci. U.S.A. 103, 3846–3851. 10.1073/pnas.0600035103 16505362 PMC1533785

[B4] Bligh E. G. Dyer W. J. (1959). A rapid method of total lipid extraction and purification. Can. J. Biochem. Physiol. 37, 911–917. 13671378 10.1139/o59-099

[B5] Boles J. R. Franks S. G. (1979). Clay diagenesis in wilcox Sandstones of Southwest Texas-implications of smectite diagenesis on sandstone cementation. J. Sediment. Petrol. 49, 55–70.

[B6] Brockman F. J. Kieft T. L. Fredrickson J. K. Bjornstad B. N. Li S. M. W. Spangenburg W.. (1992). Microbiology of vadose zone paleosols in south-central Washington state. Microb. Ecol. 23, 279–301. 24192936 10.1007/BF00164101

[B7] Brinch-Iversen J. King G. M. (1990). Effects of substrate concentration, growth state, and oxygen availability on relationships among bacterial carbon, nitrogen and phospholipid phosphorus content. FEMS Microbiol. Lett. 74, 345–355.

[B8] Cequier-Sánchez E. Rodríguez C. Ravelo A. G. Zárate R. (2008). Dichloromethane as a solvent for lipid extraction and assessment of lipid classes and fatty acids from samples of different natures. J. Agric. Food Chem. 56, 4297–4303. 10.1021/jf073471e 18505264

[B9] Chengzao J. Zheng M. Zhang Y. (2012). Unconventional hydrocarbon resources in China and the prospect of exploration and development. Petrol. Explor. Dev. 39, 139–146. 10.1016/S1876-3804(12)60026-3

[B10] Chermak J. A. Schreiber M. E. (2014). Mineralogy and trace element geochemistry of gas shales in the United States: environmental implications. Int. J. Coal Geol. 126, 32–44. 10.1016/j.coal.2013.12.005

[B11] Christie W. W. (1993). Preparation of lipid extracts from tissues. Adv. Lipid Methodol. 2, 195–213.

[B12] Cluff M. A. Hartsock A. MacRae J. D. Carter K. Mouser P. J. (2014). Temporal changes in microbial ecology and geochemistry in produced water from hydraulically fractured Marcellus Shale gas wells. Environ. Sci. Technol. 48, 6508–6517. 10.1021/es501173p 24803059

[B13] Colwell F. S. Onstott T. C. Delwiche M. E. Chandler D. Fredrickson J. K. Yao Q. J. (1997). Microorganisms from deep, high temperature sandstones: Constraints on microbial colonization. FEMS Microbiol. Rev. 20, 425–435.

[B14] Curtis J. B. (2002). Fractured shale-gas systems. Am. Assoc. Pet. Geol. Bull. 86, 1921–1938. 10.1306/61EEDDBE-173E-11D7-8645000102C1865D

[B15] D'hondt S. Jørgensen B. B. Miller D. J. Batzke A. Blake R. Cragg B. A.. (2004). Distributions of microbial activities in deep subseafloor sediments. Science 306, 2216–2221. 10.1126/science.1101155 15618510

[B16] Ewald G. Bremle G. Karlsson A. (1998). Differences between Bligh and Dyer and Soxhlet extractions of PCBs and lipids from fat and lean fish muscle: implications for data evaluation. Mar. Pollut. Bull. 36, 222–230.

[B17] Folch J. Lees M. Stanley G. H. S. (1957). A simple method for the isolation and purification of total lipides from animal tissues. J. Biol. Chem. 226, 497–509. 13428781

[B18] Franzmann P. D. Patterson B. M. Power T. R. Nichols P. D. Davis G. B. (1996). Microbial biomass in a shallow, urban aquifer contaminated with aromatic hydrocarbons: analysis by phospholipid fatty acid content and composition. J. Appl. Bacteriol. 80, 617–625. 8698663 10.1111/j.1365-2672.1996.tb03266.x

[B19] Fredricks H. F. Hinrichs K.-U. (2007). Data report: intact membrane lipids as indicators of subsurface life in cretaceous and paleogene sediments from sites 1257 and 1258, in Proceedings of ODP, Scientific Results, Vol. 207 (College Station, TX: Ocean Drilling Program), 1–11.

[B20] Fredrickson J. K. McKinley J. P. Bjornstad B. N. Long P. E. Ringelberg D. B. White D. C. (1997). Pore-size constraints on the activity and survival of subsurface bacteria in a late cretaceous shale-sandstone sequence, northwestern New Mexico. Geomicrobiol. J. 14, 183–202.

[B21] Frostegard A. Baath E. (1996). The use of phospholipid fatty acid analysis to estimate bacterial and fungal biomass in soil. Biol. Fertil. Soils 22, 59–65.

[B22] Frostegard A. Tunlid A. Baath E. (1991). Microbial biomass measured as total lipid phosphate in soils of different organic content. J. Microbiol. Methods 14, 151–163.

[B23] Gaspar J. Mathieu J. Yang Y. Tomson R. Leyris J. D. Gregory K. B. (2014). Microbial dynamics and control in shale gas production. Environ. Sci. Technol. Lett. 1, 465–473. 10.1021/ez5003242

[B24] Ginn T. R. Scheibe T. D. Murphy E. M. DeFlaun M. F. Onstott T. C. (1998). Effects of chemical heterogeneity on subsurface fate and transport involving biotic reaction systems: two examples, in American Geophysical Union Fall Meeting, (San Francisco, CA) December 1998. Eos Trans 79:F294.

[B25] Glusker J. P. (1980). Citrate conformation and chelation: enzymic implications. Acc. Chem. Res. 13, 345–352.

[B26] Gómez-Brandón M. Lores M. Dominguez J. (2008). Comparison of extraction and derivatization methods for fatty acid analysis in solid environmental matrixes. Anal. Bioanal. Chem. 392, 505–514. 10.1007/s00216-008-2274-7 18651136

[B27] Gómez-Brandón M. Lores M. Domínguez J. (2010). A new combination of extraction and derivatization methods that reduces the complexity and preparation time in determining phospholipid fatty acids in solid environmental samples. Bioresour. Technol. 101, 1348–1354. 10.1016/j.biortech.2009.09.047 19800785

[B28] Guckert J. B. Antworth C. P. Nichols P. D. White D. C. (1985). Phospholipid, ester-linked fatty-acid profiles as reproducible assays for changes in prokaryotic community structure of estuarine sediments. FEMS Microbiol. Ecol. 31, 147–158.

[B29] Haldeman D. L. Amy P. S. Ringelberg D. White D. C. (1993). Characterization of the microbiology within a 21 m 3 section of rock from the deep subsurface. Microb. Ecol. 26, 145–159. 24190010 10.1007/BF00177049

[B30] Haldeman D. L. Amy P. S. White D. C. Ringelberg D. B. (1994). Changes in bacteria recoverable from subsurface volcanic rock samples during storage at 4 C. Appl. Environ. Microbiol. 60, 2697–2703. 16349343 10.1128/aem.60.8.2697-2703.1994 PMC201711

[B31] Haldeman D. L. Amy P. S. Ringelberg D. White D. C. Garen R. E. Ghiorse W. C. (1995). Microbial growth and resuscitation alter community structure after perturbation. FEMS Microbiol. Ecol. 17, 27–37. 10.1111/j.1574-6941.1995.tb00124.x

[B32] Inagaki F. Hinrichs K.-U. Kubo Y. The IODP Expedition, 337 Scientists (2016). IODP Expedition 337: deep coalbed biosphere off shimokita–microbial processes and hydrocarbon system associated with deeply buried coalbed in the ocean, Sci. Dril. 21, 17–28. 10.5194/sd-21-17-2016

[B33] Javadpour F. (2009). Nanopores and apparent permeability of gas flow in mudrocks (shales and siltstone). J. Can. Petrol. Technol. 48, 16–21. 10.2118/09-08-16-DA

[B34] Kalb E. Frey S. Tamm L. K. (1992). Formation of supported planar bilayers by fusion of vesicles to supported phospholipid monolayers. Biochim. Biophys. Acta 1103, 307–316. 1311950 10.1016/0005-2736(92)90101-q

[B35] Kieft T. L. Ringelberg D. B. White D. C. (1994). Changes in ester-linked phospholipid fatty-acid profiles of subsurface bacteria during starvation and desiccation in a porous-medium. Appl. Environ. Microbiol. 60, 3292–3299. 16349382 10.1128/aem.60.9.3292-3299.1994 PMC201801

[B36] Krumholz L. R. McKinley J. P. Ulrich G. A. Suflita J. M. (1997). Confined subsurface microbial communities in Cretaceous rock. Nature 386, 64–66. 10.1038/386064a0

[B37] Lahann R. W. Campbell R. C. (1980). Adsorption of palmitic acid on calcite. Geochim. Cosmochim. Acta 44, 629–634.

[B38] Lazar I. Petrisor I. G. Yen T. F. (2007). Microbial enhanced oil recovery (MEOR). Pet. Sci. Technol. 25, 1353–1366. 10.1080/10916460701287714

[B39] Lee H. Y. Shin S. H. R. Drews A. M. Chirsan A. M. Lewis S. A. Bishop K. J. (2014). Self-assembly of nanoparticle amphiphiles with adaptive surface chemistry. ACS Nano 8, 9979–9987. 10.1021/nn504734v 25229312

[B40] Lengger S. K. Hopmans E. C. Sinninghe Damsté J. S. Schouten S. (2012). Comparison of extraction and work up techniques for analysis of core and intact polar tetraether lipids from sedimentary environments. Org. Geochem. 47, 34–40. 10.1016/j.orggeochem.2012.02.009

[B41] Lopez-Avila V. (1999). Sample preparation for environmental analysis. Crit. Rev. Anal. Chem. 29, 195–230.

[B42] Lopez-Avila V. Young R. Beckert W. F. (1994). Microwave-assisted extraction of organic compounds from standard reference soils and sediments. Anal. Chem. 66, 1097–1106.

[B43] Lopez-Avila V. Young R. Benedicto J. Ho P. Kim R. Beckert W. F. (1995). Extraction of organic pollutants from solid samples using microwave energy. Anal. Chem. 67, 2096–2102. 10.1021/ac00109a031

[B44] Lores M. Gómez-Brandón M. Pérez-Díaz D. Domínguez J. (2006). Using FAME profiles for the characterization of animal wastes and vermicomposts. Soil Biol. Biochem. 38, 2993–2996. 10.1016/j.soilbio.2006.05.001

[B45] McMahon S. Parnell J. (2014). Weighing the deep continental biosphere. FEMS Microbiol. Ecol. 87, 113–120. 10.1111/1574-6941.12196 23991863

[B46] Meyers P. A. Quinn J. G. (1973). Factors affecting association of fatty-acids with mineral particles in sea-water. Geochim. Cosmochim. Acta 37, 1745–1759.

[B47] Mohan A. M. Hartsock A. Hammack R. W. Vidic R. D. Gregory K. B. (2013). Microbial communities in flowback water impoundments from hydraulic fracturing for recovery of shale gas. FEMS Microbiol. Ecol. 86, 567–580. 10.1111/1574-6941.12183 23875618

[B48] Morris R. J. Calvert S. E. (1975). Fatty-acid uptake by marine sediment particles. Geochim. Cosmochim. Acta 39, 377–381.

[B49] Murphy E. M. Ginn T. R. Chilakapati A. Resch C. T. Phillips J. L. Wietsma T. W. (1997). The influence of physical heterogeneity on microbial degradation and distribution in porous media. Water Resour. Res. 33, 1087–1103.

[B50] Nielsen P. Petersen S. O. (2000). Ester-linked polar lipid fatty acid profiles of soil microbial communities: a comparison of extraction methods and evaluation of interference from humic acids. Soil Biol. Biochem. 32, 1241–1249. 10.1016/S0038-0717(00)00041-9

[B51] Oksanen F. J. Guillaume B. Roeland K. Pierre Legendre Peter R. Minchin R. B. O'Hara Gavin L.. (2016). Community Ecology Package. R package version 2.3-5 Year: 2016. Available online at: https://CRAN.R-project.org/package=vegan

[B52] Oleson T. A. Sahai N. Pedersen J. A. (2010). Electrostatic effects on deposition of multiple phospholipid bilayers at oxide surfaces. J. Colloid Interface Sci. 352, 327–336. 10.1016/j.jcis.2010.08.057 20869065

[B53] Onstott T. C. Phelps T. J. Colwell F. S. Ringelberg D. White D. C. Boone D. R. (1998). Observations pertaining to the origin and ecology of microorganisms recovered from the deep subsurface of Taylorsville Basin, Virginia. Geomicrobiol. J. 15, 353–385.

[B54] Papadopoulou E. S. Karpouzas D. G. Menkissoglu-Spiroudi U. (2011). Extraction parameters significantly influence the quantity and the profile of PLFAs extracted from soils. Microb. Ecol. 62, 704–714. 10.1007/s00248-011-9863-2 21556882

[B55] Passey Q. R. Bohacs K. Esch W. L. Klimentidis R. Sinha S. (2010). From oil-prone source rock to gas-producing shale reservoir-geologic and petrophysical characterization of unconventional shale gas reservoirs, in International Oil and Gas Conference and Exhibition in China. Society of Petroleum Engineers.

[B56] Pfiffner S. M. Cantu J. M. Smithgall A. Peacock A. D. White D. C. Moser D. P. (2006). Deep subsurface microbial biomass and community structure in Witwatersrand Basin mines. Geomicrobiol. J. 23, 431–442. 10.1080/01490450600875712

[B57] Renaud S. M. Thinh L. V. Parry D. L. (1999). The gross chemical composition and fatty acid composition of 18 species of tropical Australian microalgae for possible use in mariculture. Aquaculture 170, 147–159.

[B58] Ringelberg D. B. Davis J. D. Smith G. A. Pfiffner S. M. Nichols P. D. Nickels J. S. (1989). Validation of signature polarlipid fatty acid biomarkers for alkane-utilizing bacteria in soils and subsurface aquifer materials. FEMS Microbiol. Ecol. 5, 39–50.

[B59] Ringelberg D. B. Sutton S. White D. C. (1997). Biomass, bioactivity and biodiversity: microbial ecology of the deep subsurface: analysis of ester-linked phospholipid fatty acids. FEMS Microbiol. Rev. 20, 371–377.

[B60] Rogner H. H. (1997). An assessment of world hydrocarbon resources. Ann. Rev. Energy Environ. 22, 217–262.

[B61] Sahai N. Kaddour H. Dalai P. Wang Z. Bass G. Gao M. (2017). Mineral surface chemistry and nanoparticle-aggregation control membrane self-assembly. Sci. Rep. 7:43418. 10.1038/srep43418 28266537 PMC5339912

[B62] Schippers A. Neretin L. N. (2006). Quantification of microbial communities in near-surface and deeply buried marine sediments on the Peru continental margin using real-time PCR. Environ. Microbiol. 8, 1251–1260. 10.1111/j.1462-2920.2006.01019.x 16817933

[B63] Sharma S. Mulder M. L. Sack A. Schroeder K. Hammack R. (2014). Isotope approach to assess hydrologic connections during Marcellus Shale drilling. Groundwater 52, 424–433. 10.1111/gwat.12083 23772970

[B64] Shaw D. B. Weaver C. E. (1965). The mineralogical composition of shale. J. Sediment. Petrol. 35, 213–222.

[B65] Sondergeld C. H. Ambrose R. J. Rai C. S. Moncrieff J. (2010). Micro-structural studies of gas shales, in SPE Unconventional Gas Conference. Pittsburgh, PA: Society of Petroleum Engineers.

[B66] Stevens M. J. Donato L. J. Lower S. K. Sahai N. (2009). Oxide-dependent adhesion of the jurkat Line of T Lymphocytes. Langmuir 25, 6270–6278. 10.1021/la8040192 19341241

[B67] Thomas M. M. Clouse J. A. Longo J. M. (1993). Adsorption of organic compounds on carbonate minerals. Chem. Geol. 109, 201–213.

[B68] Vetter W. Weichbrodt M. Hummert K. Glotz D. Luckas B. (1998). Combined microwave-assisted extraction and gel permeation chromatography for the determination of chlorinated hydrocarbons in seal blubber and cod livers. Chemosphere 37, 2439–2449. 9828347 10.1016/s0045-6535(98)00300-2

[B69] White D. C. Davis W. M. Nickels J. S. King J. D. Bobbie R. J. (1979). Determination of the sedimentary microbial biomass by extractable lipid phosphate. Oecologia 40, 51–62. 28309603 10.1007/BF00388810

[B70] White D. C. Ringelberg D. B. (1998). Signature Lipid Biomarker Analysis, Vol. 255 New York, NY: Oxford University Press.

[B71] Whitman W. B. Coleman D. C. Wiebe W. J. (1998). Prokaryotes: the unseen majority. Proc. Natl. Acad. Sci. U.S.A. 95, 6578–6583. 9618454 10.1073/pnas.95.12.6578 PMC33863

[B72] Wilkins M. J. Daly R. A. Mouser P. J. Trexler R. Sharma S. Cole D. R.. (2014). Trends and future challenges in sampling the deep terrestrial biosphere. Front. Microbiol. 5:481. 10.3389/fmicb.2014.00481 25309520 PMC4162470

[B73] Wu Y. Ding N. Wang G. Xu J. Wu J. Brookes P. C. (2009). Effects of different soil weights, storage times and extraction methods on soil phospholipid fatty acid analyses. Geoderma 150, 171–178. 10.1016/j.geoderma.2009.02.003

[B74] Xu L. Davis T. A. Porter N. A. (2009). Rate constants for peroxidation of polyunsaturated fatty acids and sterols in solution and in liposomes. J. Am. Chem. Soc. 131, 13037–13044. 10.1021/ja9029076 19705847 PMC3656724

[B75] Young J. C. (1995). Microwave-assisted extraction of the fungal metabolite ergosterol and total fatty acids. J. Agric. Food Chem. 43, 2904–2910.

[B76] Zhang C. L. Pancost R. D. Sassen R. Qian Y. Macko S. A. (2003). Archaeal lipid biomarkers and isotopic evidence of anaerobic methane oxidation associated with gas hydrates in the Gulf of Mexico. Org. Geochem. 34, 827–836. 10.1016/S0146-6380(03)00003-2

[B77] Zhang C. Palumbo A. V. Phelps T. J. Beauchamp J. J. Brockman F. J. Murray C. J. (1998). Grain size and depth constraints on microbial variability in coastal plain subsurface sediments. Geomicrobiol. J. 15, 171–185.

[B78] Zullig J. J. Morse J. W. (1988). Interaction of organic-acids with carbonate mineral surfaces in seawater and related solutions.1. Fatty-Acid Adsorption. Geochim. Cosmochim. Acta 52, 1667–1678.

