# Corrigendum: Modified Lipid Extraction Methods for Deep Subsurface Shale

**DOI:** 10.3389/fmicb.2017.02141

**Published:** 2017-10-24

**Authors:** Rawlings N. Akondi, Ryan V. Trexler, Susan M. Pfiffner, Paula J. Mouser, Shikha Sharma

**Affiliations:** ^1^Department of Geology and Geography, West Virginia University, Morgantown, WV, United States; ^2^Civil, Environmental and Geodetic Engineering, The Ohio State University, Columbus, OH, United States; ^3^Center for Environmental Biotechnology, University of Tennessee, Knoxville, TN, United States

**Keywords:** PLFA, DGFA, microbial biomass, deep subsurface, shale ecosystem

In the original article, we neglected to include the funder Department of Energy's National Energy Technology Laboratory (DOE-NETL), DE# FE0024297 to SS and PJM. The authors apologize for this error and state that this does not change the scientific conclusions of the article in any way.

## Conflict of interest statement

The authors declare that the research was conducted in the absence of any commercial or financial relationships that could be construed as a potential conflict of interest.

