# Supplementary figures and images for: Modified Lipid Extraction Methods for Deep Subsurface Shale
Source: Front Microbiol. 2017 Jul 25;8:1408. doi: 10.3389/fmicb.2017.01408 (PMC5524817; doi:10.3389/fmicb.2017.01408)

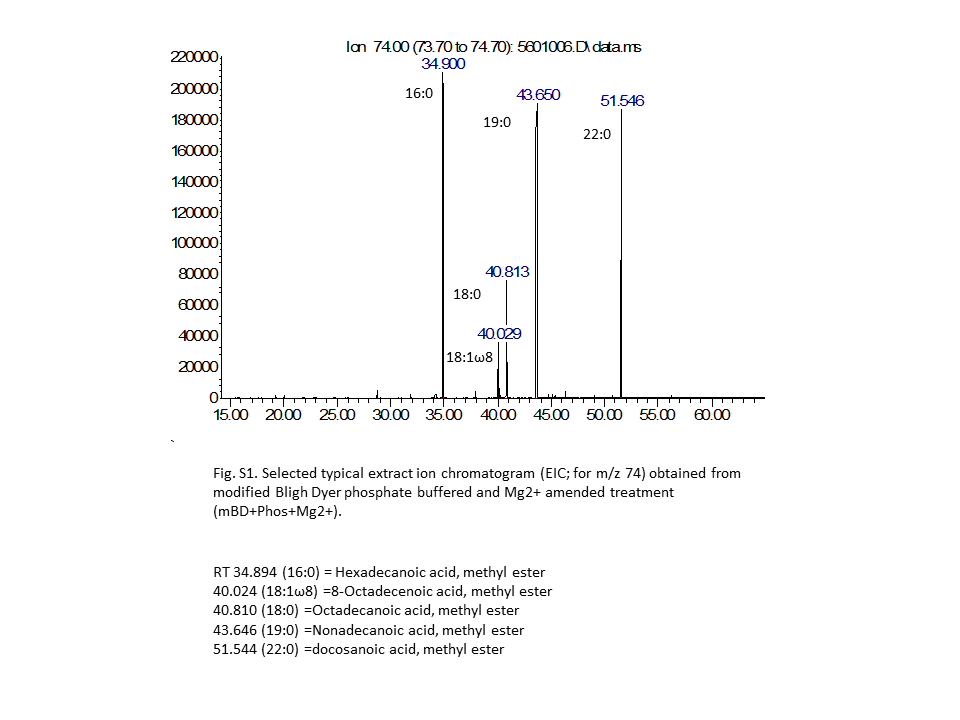

Supplement: Supplementary file 1 [file Image1.TIF]

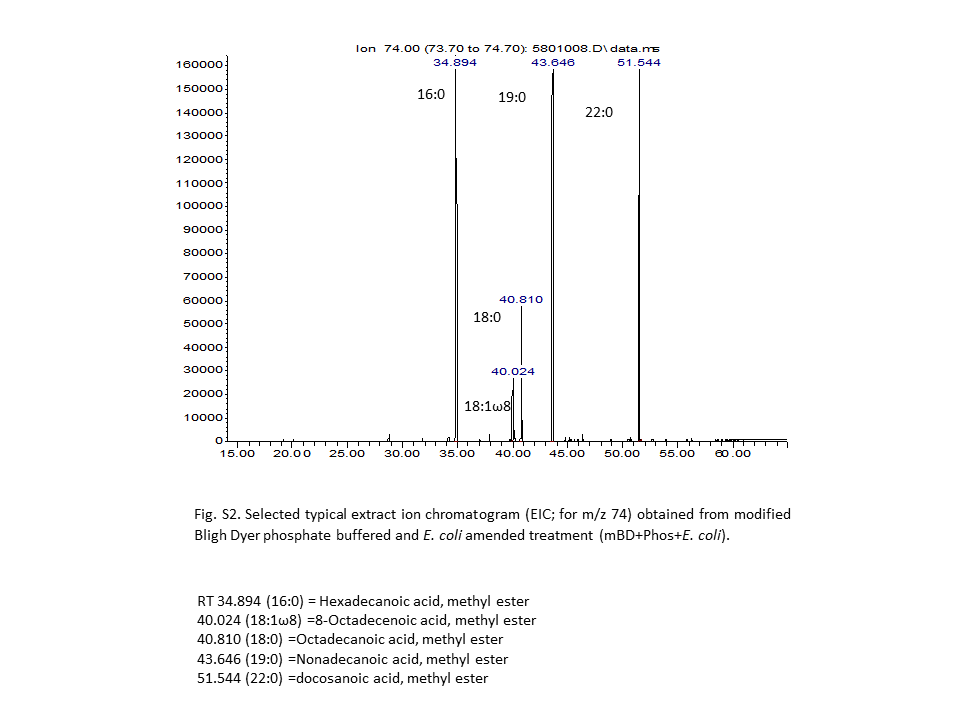

Supplement: Supplementary file 2 [file Image2.TIF]

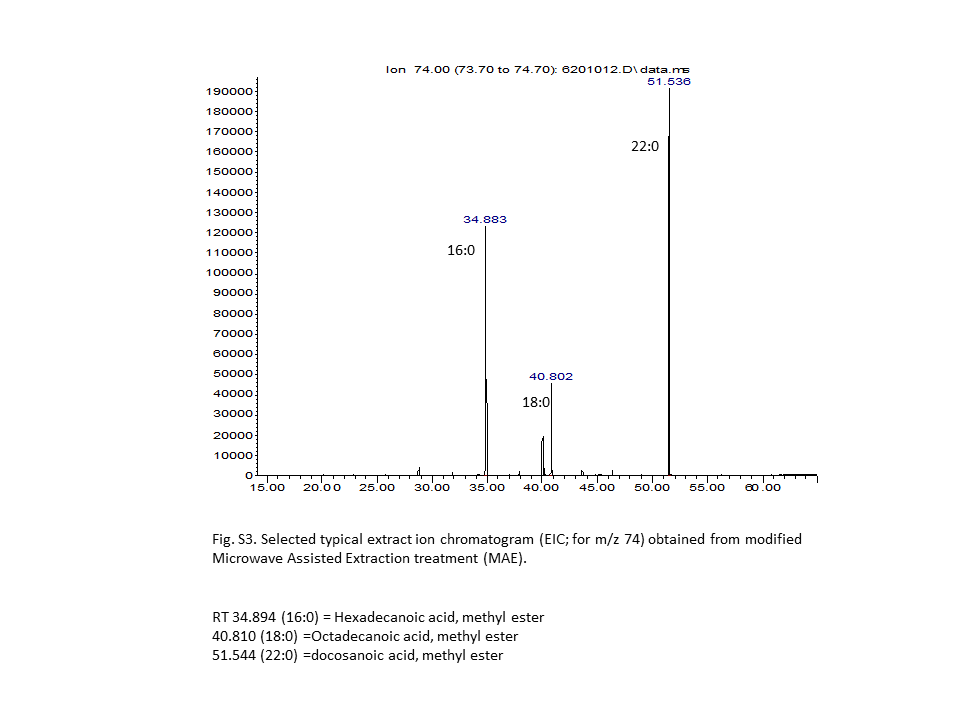

Supplement: Supplementary file 3 [file Image3.TIF]

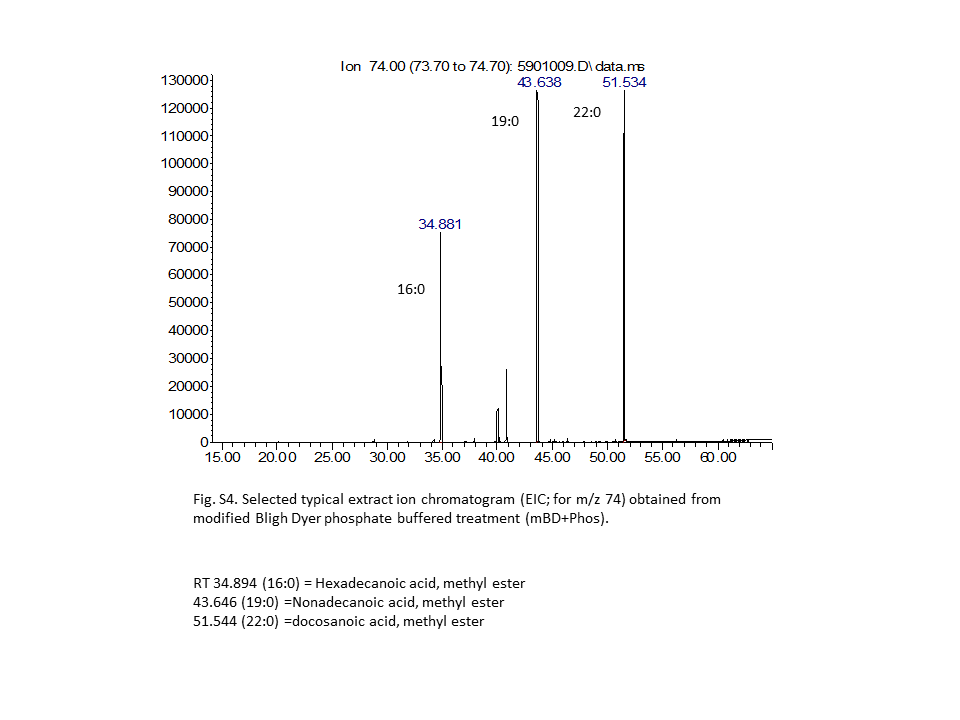

Supplement: Supplementary file 4 [file Image4.TIF]

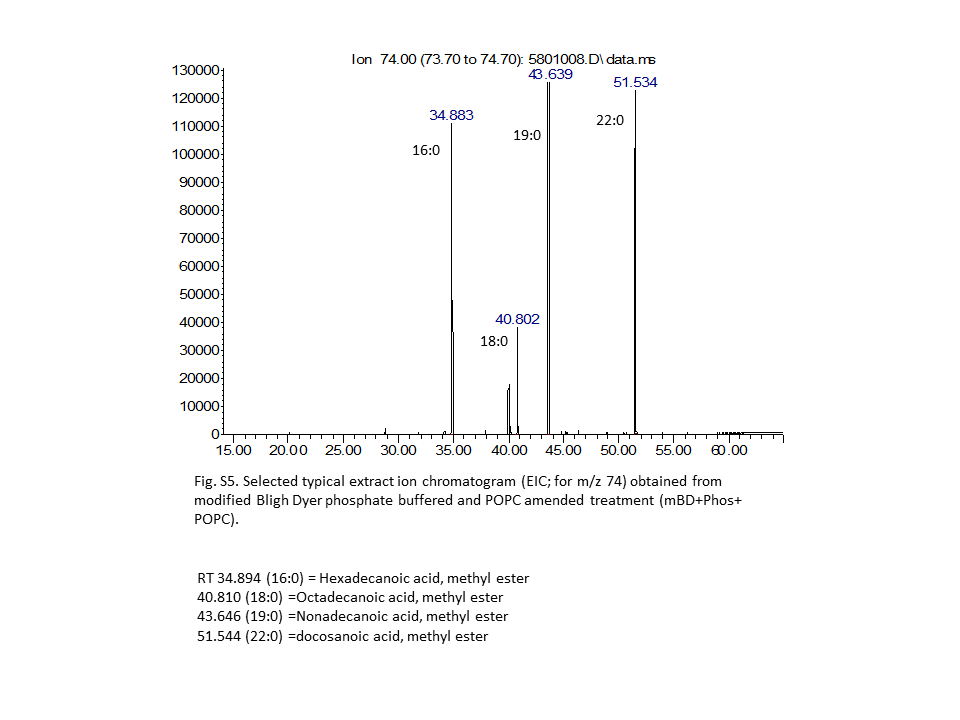

Supplement: Supplementary file 5 [file Image5.TIF]
